# Referral pathway and competency profiles of primary care physiotherapists and kinesiologists for physical activity interventions for diabetes: a modified Delphi study

**DOI:** 10.1186/s12875-024-02611-1

**Published:** 2024-10-15

**Authors:** Carly Proctor, Cara L. Brown

**Affiliations:** The College of Rehabilitation Sciences, Rady Faculty of Health Sciences, University of Manitoba, Winnipeg, MB Canada

**Keywords:** Diabetes, Physical Activity, Exercise, Physiotherapist, Kinesiologist, Competencies, Referral Pathway, Delphi, Primary Care

## Abstract

**Background:**

High quality diabetes care is an essential service in primary care settings since the prevalence and associated complications of diabetes is increasing. Physical activity is effective for the prevention and management of diabetes yet is underutilized in diabetes care. Exercise professionals have specialized skills to deliver physical activity interventions, but effective interprofessional collaboration for diabetes care requires role clarity. This study established the competencies of entry-level physiotherapists and kinesiologists for physical activity interventions for diabetes care in primary care settings and used these competencies to develop clinical tools to promote role clarity in interprofessional care teams.

**Methods:**

We used a modified Delphi process. Eleven physiotherapy and three kinesiology subject matter experts participated in two rounds of Delphi surveys to develop discipline and context specific competencies. These competencies were used to draft competency profiles and a referral pathway tool. Eleven of the participants then participated in a focus group for member-checking of the tools. Descriptive statistics and content analysis were used to analyze quantitative and qualitative data respectively.

**Results:**

The modified Delphi process resulted in 38 physiotherapy and 27 kinesiology competencies that identify the distinct roles of physiotherapists and kinesiologists in delivering physical activity interventions for diabetes care. The physiotherapy competencies describes their unique role in supporting people with all types of diabetes to engage in physical activity despite complex medical or physical barriers. The kinesiology competencies indicate where these professionals may require additional training, especially when working with people living with type 1 diabetes or who are pregnant. All developed tools had good face validity and were seen to be potentially useful tools by the subject matter experts.

**Conclusions:**

The findings highlight that both physiotherapists and kinesiologists have fundamental skills and abilities to deliver physical activity interventions to people living with diabetes, but that different exercise professionals may be needed depending on the complexity of the clinical profile. The developed clinical tools support improved interprofessional collaboration by clarifying physiotherapy and kinesiology roles in delivering physical activity interventions for diabetes care and highlighting how the two distinct professions can contribute to addressing the growing diabetes epidemic in primary care.

**Supplementary Information:**

The online version contains supplementary material available at 10.1186/s12875-024-02611-1.

## Background

Diabetes mellitus is a growing epidemic in Canada with nearly one third of Canadians living with diabetes or prediabetes ​[1]​. The rising rates of diabetes, and their associated comorbidities, is driving the demand for more complex needs to be addressed in primary care settings [2]​. Clinical outcomes for people living with diabetes indicate that providers are struggling to meet treatment targets [3].​ Failure to meet these targets have well-recognized negative health implications on patient health and disability [4]​​.

Exercise is known to be a highly effective treatment for the prevention and management of diabetes [5–9]​, but is underutilized in primary care settings [10, 11]. Exercise has the potential to improve many of the outcomes not currently being met in primary care settings, like glycemic control [8]​, blood pressure and lipid profiles [5, 7]. Exercise interventions have also been shown to improve body weight, insulin sensitivity, decrease medication and insulin use [5, 7]​, and reduce or prevent vascular complications that occur with diabetes [6, 7]. Despite being a pillar of diabetes prevention and management, physical activity is one of the most underutilized in primary care settings with nearly half of all Canadians being inactive [12].

Quality physical activity interventions are a collaborative interaction between a health care provider and the person living with diabetes, with the aim to address facilitators and barriers to engaging in this self-care behavior [7]. In this paper, we are using the term physical activity interventions to encompass the wide scope of ways health professionals can support individuals to engage in physical activity and provide self-management education and support. Dependent on the individual’s goals and needs, physical activity intervention activities may include, individualized or group education, precaution or contraindication screening, behavior change counselling, structured and/or supervised physical activity, resource navigation, and/or other clinical support.

Most people living with diabetes have at least one other health concern or medical condition such as overweight or obese (86%), hypertension (64%), macrovascular disease (26%) and microvascular disease (25%) [3]. These comorbidities, insulin use, and/or pregnancy can make attaining or working towards physical activity recommendations more challenging for people living with diabetes and add complexity to the design and delivery of physical activity interventions by primary care providers [13–15]​. Primary care providers are currently struggling to support safe and effective physical activity for people living with diabetes [16, 17]. These providers are typically physicians and diabetes educators (primarily pharmacists, dietitians, and nurses with additional training), who feel unprepared to offer physical activity interventions safely and effectively to patients using insulin or with more complex disease presentations [10, 11, 17].

A potential solution to this gap in care is the inclusion and integration of exercise specialists like physiotherapists and kinesiologists in primary care teams. These exercise specialists have expertise in physical activity intervention delivery and thus may be better positioned to support physical activity behaviors. Clinical Practice Guidelines (CPG) for diabetes care acknowledges the complexity of physical activity interventions and the potential risks to people living with diabetes when starting or engaging in physical activity but fall short of providing clear guidance regarding what type of health care professional could support primary care teams in providing this important aspect of diabetes care [7]. For nutrition therapy and pharmacological therapy, the inclusion of dietitians and pharmacists are recommended, along with expansion of professional roles [18]. Yet, regarding physical activity, the CPGs have only a small number of references prompting providers to consider a referral to an *exercise specialist* or *qualified trainer* [7] which provides no specificity for primary care team members regarding qualifications.

Ambiguity regarding the qualifications of exercise professionals is common. Many exercise intervention studies use terms such as exercise specialists for the person delivering the exercise intervention [5, 19]. Often this term is not defined and personal trainers, who have completed a short training process, are not necessarily differentiated from university educated professionals with wide-ranging educational backgrounds and expertise such as exercise physiologists, physiotherapists and, kinesiologists [20]. Physical activity referral programs, also known as Physical Activity Referral Schemes (PARS), or Exercise Referral Schemes (ERS) have been in use in Europe since the 1990’s [21]. These referral programs, where primary care providers refer sedentary individuals to a third-party service for an exercise program have had underwhelming results with low uptake and adherence [22–24]. However, a systematic review of 27 PARS studies from eight countries found that exercise interventions that included support from physiotherapists and exercise physiologists improved adherence and patient outcomes highlighting that professional specificity may be important [21].

Having more exercise specialists in primary care has great potential for improving diabetes outcomes. However, a common challenge to providing effective interprofessional care is poorly defined professional roles that impede care coordination and collaboration [25]. Primary care team members need to have a strong understanding of the role and competencies of exercise professionals in primary care diabetes care to work with them and generate physical activity referrals effectively [25, 26]. This study established the competencies of entry-level physiotherapists and kinesiologists in physical activity intervention delivery for diabetes care in primary care settings and then used these competencies to develop clinical tools to support quality team delivery of diabetes care.

## Methods

We used a modified Delphi method where subject matter experts developed discipline and context specific competencies through two rounds of Delphi surveys and a focus group. These competencies were used to draft three clinical tools: 1) a physiotherapy competency profile, 2) a kinesiology competency profile and, 3) a referral pathway for physical activity intervention for diabetes.

The Delphi method is a well-established technique [27, 28] where the consensus of experts is used to increase understanding on a topic where information is contradictory or lacking [27–29]. Key features of the Delphi method are obtaining expert opinion through iterative rounds and providing anonymized, controlled feedback. Controlled feedback is the process of providing participants a summary of the group’s responses from the previous round, allowing each participant the opportunity to reconsider and/or clarify their opinion on the issue [27].

**Fig. 1:**
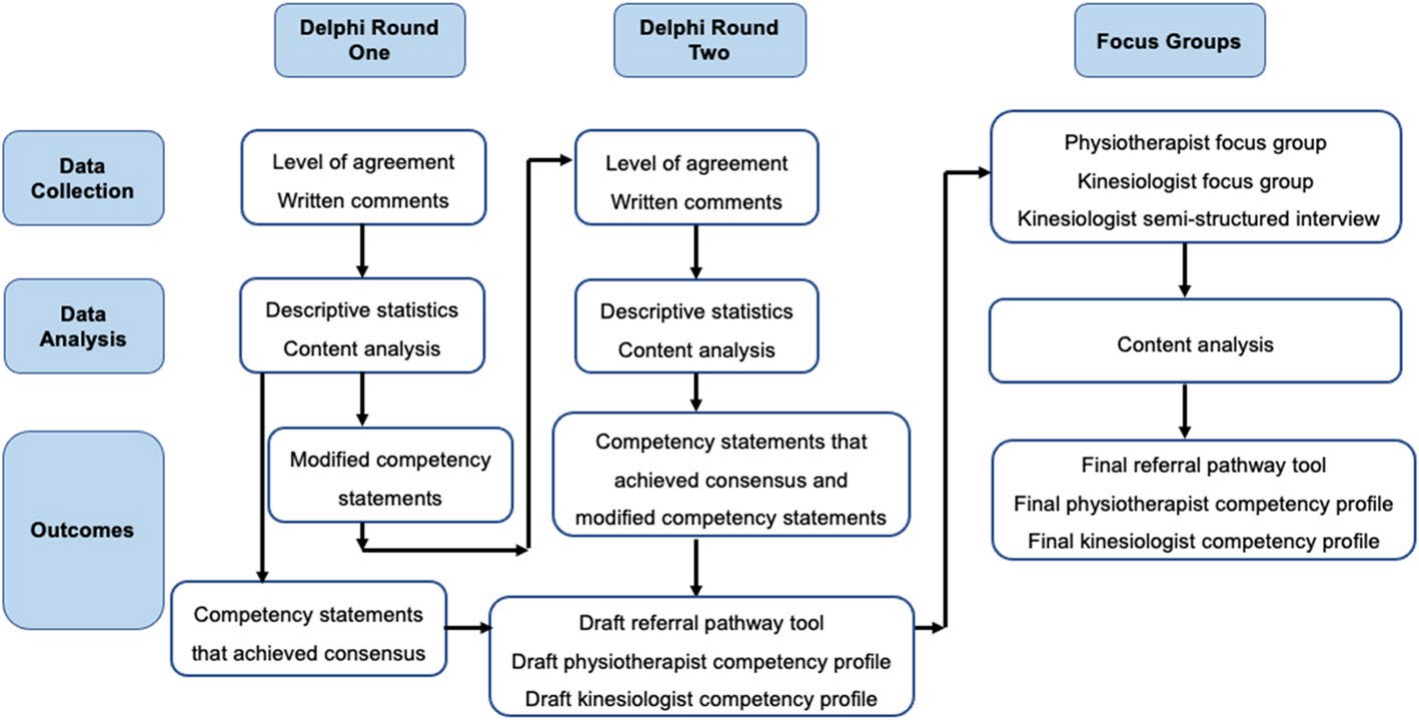
Data collection and analysis stages

Modifications to the Delphi are common and accepted in the literature [29], and we used two primary modifications in our method. An overview of our data collection and analysis method is displayed in Fig. 1. The first modification was the addition of focus groups following two Delphi rounds as a way to clarify and increase confidence in the Delphi survey findings [30]. The second modification was that we adapted existing national competency profiles [31, 32] to present in the first Delphi round, rather than asking participants to generate the competencies from the ground up.

### Recruitment and sampling

Recruitment occurred between April and June 2022. Participants were targeted for their expertise in diabetes management using purposive and snowball sampling of physiotherapy and kinesiology experts. Given the emerging role of exercise specialists in primary care, the number of expert clinicians working in diabetes care is limited. Considering this and the benefits of heterogenous sampling [27, 28], clinicians, academics and researchers were included in our recruitment strategy. Physiotherapists and kinesiologists were recruited via email through 17 Canadian university programs, eight professional organizations and by direct email to 12 subject matter experts. To find the latter subject matter experts, we did an online search using google and university websites for physiotherapists or kinesiologists who were researchers and/or Certified Diabetes Educators (CDE)® who indicated in their online profile an interest or experience in physical activity interventions for diabetes care. Of those, we emailed individuals who had publicly available contact information. Invitations to participate were also posted on the primary investigator’s (CP) social media pages and shared with national and provincial professional organizations.

### Eligibility criteria

Participants were English speaking physiotherapists or kinesiologists registered with a Canadian provincial regulatory body or affiliated with the Canadian Kinesiology Alliance (CKA). Participants were eligible for the study if they met one of the following criteria: 1) certified with the Canadian Diabetes Educator Certification Board as a Certified Diabetes Educator (CDE)® within the last 5 years, 2) an academic or researcher with expertise in diabetes, or 3) employed for 2 or more years in primary care clinics or specialty clinics (amputees, renal, endocrinology) with self-reported high prevalence of clients with diabetes.

### Demographics

Participant characteristics are displayed in Table 1. Most of the 11 physiotherapy participants were working as clinicians (91%), most in primary care settings (73%), with most having 10 or more years of clinical experience (90%) and over half with more than 20 years’ experience (54%). Clinical experience ranged from large urban settings to remote northern Indigenous communities. There was also representation from academics and researchers (18%). Ancestry was described as White or European (6), Canadian Métis (1), Philippines (1), South Asian (1), and two participants chose not to answer this question. Kinesiology participants included clinicians (67%) and academics/researchers (33%). All three kinesiologist participants described their ancestry as White.

**Table 1:** Demographic information of participants

Characteristics	Physiotherapists *n* = 11 n (%)	Kinesiologists *n* = 3 n (%)
Highest degree obtained		
Bachelor	7 (64)	2 (67)
Doctorate (PhD)	2 (18)	1 (33)
Research-based master	2 (18)	
Other certifications^a^		
Certified Diabetes Educator (CDE)		1 (33)
Clinical Exercise Physiologist (CEP)		1 (33)
Current work^a^		
Clinicians	10 (91)	2 (67)
Academic/researcher	2 (18)	1 (33)
Clinical practice setting^a^		
Primary care	8 (73)	1 (33)
Specialty clinic	2 (18)	
Other setting	4 (36)	1 (33)
Experience as a clinician		
5–10 years	1 (9)	2 (67)
10–20 years	4 (36)	1 (33)
> 20 years	6 (54)	
Experience as an academic		
< 1 year	1 (9)	
10–20 years	2 (18)	
> 20 years		1 (33)
Experience as a researcher		
< 1 year	1 (9)	
1–10 years	3 (27)	1 (33)
10–20 years	1 (9)	
> 20 years		1 (33)
Gender^b^		
Female	7 (64)	2 (67)
Male	2 (18)	1 (33)
Did not answer	2 (18)	
Province/Territory		
Manitoba	9 (82)	
Nova Scotia	1 (9)	1 (33)
Alberta	1 (9)	1 (33)
Ontario		1 (33)

^a^Participants could choose all that applies

^b^Gender data was collected using an open text box

### Process for developing diabetes care competencies for Delphi round 1

The initial list of diabetes related competency statements for participants to consider in the first Delphi round was modified from the National Physiotherapy Advisory Group’s (NPAG) *Competency profile for physiotherapists in Canada* [33]. The NPAG’s competency profile uses seven practice domains (physical therapy expertise, communication, collaboration, management, leadership, scholarship, and professionalism) to categorize 34 entry-to-practice competencies of physiotherapists. Each of the NPAG competencies have two or more *entry-to-practice milestones* which are specific abilities related to that competency that is expected of an entry-level physiotherapist. These milestones were the basis for the Delphi round one survey.

To ensure the competency statements developed for this study were specific to physical activity interventions for diabetes care and specific to the exercise professional (physiotherapist or kinesiologist), modifications of the milestones were informed by:

- Diabetes Canada’s Clinical Practice Guidelines (CPGs) [31] which are evidence-based guidelines intended to guide clinical practice in diabetes prevention and management,
- The Canadian Diabetes Educator Certification Board’s CDE® competency profile [34] which is the basis for the CDE® certification exam in Canada and encompasses the disease-specific competencies needed to meet the broad care needs of people living with diabetes, and
- The Canadian Kinesiology Association’s competency profile for entry-level kinesiologists [32] which consists of 54 competencies across five domains: knowledge, kinesiology practical experience, professionalism/professional practice, communication and collaboration, and professional development.

We reviewed all the NPAG milestones and extracted those that were relevant to physical activity interventions for diabetes care in primary care settings. We modified these relevant milestones into draft competency statements that reflected the intervention, population, and clinical context of this study. At times, this involved combining similar milestones to create fewer, more comprehensive statements, or expanding one milestone into multiple competency statements to add specificity. Examples are provided in Table 2.

**Table 2:** Examples of adapting NPAG milestones for Delphi round 1 survey

NPAG milestone	Draft physiotherapist competency statement
6.5 Contribute to the education of peers and other healthcare providers	Identify the learning needs of other healthcare providers related to physical activity and contribute to and assess the effectiveness of learning activities
6.5 Identify the physiotherapy-related learning needs of others	
6.5 Assess effectiveness of learning activities	
1.2 Identify client specific precautions, contraindications and risks	Identifies client-specific precautions, contraindications and risks to physical activity participation from acute hyperglycemia, hypoglycemia or pseudo-hypoglycemia
	Identifies client-specific precautions, contraindications and risks to physical activity participation from diabetes related comorbidities (e.g., foot ulcer, pre-proliferative retinopathy, autonomic neurological dysfunction)
	Identifies client-specific precautions, contraindications and risks to physical activity participation from non-diabetes related comorbidities in people living with diabetes

We developed 40 physiotherapy and 29 kinesiology competencies organized in four domains from the initial 140 NPAG milestones from seven domains for the first Delphi survey round. The Delphi surveys were pilot tested for both clarity and functionality and minor changes were made based on the pilot participants’ feedback. The round 1 Delphi surveys for physiotherapy and kinesiology are available in Additional files 1 and 2 respectively.

### Data collection and analysis

Data collection and analysis was iterative. To promote clarity, we describe it here in chronological order of activities including results that informed subsequent stages of the study: 1) Delphi round 1 data collection and analysis, 2) Delphi round 2 data collection and analysis, 3) draft clinical tool development for presentation at the focus groups, 4) focus group data collection and analysis. Research Electronic Data Capture (REDCap) [35], a web-based survey platform, was used to administer the two rounds of Delphi surveys and a demographic questionnaire. Participants were emailed a link to the REDCap survey and had two weeks to complete each survey. Email reminders were sent out one week, two days and one day prior to the deadlines.

#### Delphi survey round 1

Participants rated their level of agreement with each of the draft competency statements using a five-point Likert scale (strongly agree [1] to strongly disagree [5]). Likert scale data were analyzed at the ordinal level by means of descriptive statistics using median level of agreement and range for each competency statement. Following guidance from other health care competency and referral pathway studies [36, 37], consensus was considered established if 80% of participants agreed with the statement (rating their level of agreement as '1 and 2’ on the five-point Likert scale) or disagreed with the statement (rating agreement as ‘4 and 5’).

A comment box followed each competency statement so that participants could suggest modifications to the competency statement, identify any missing competencies or add anything else they would like to share. Written comments were analyzed using content analysis as described by Elo and Kyngäs [38]. After reading through all the experts’ comments, data within each competency statement was organized into categories by grouping similar ideas and concepts. Each idea/concept was considered and determined if it indicated the competency statement needed to be modified or eliminated for the following round. These decisions also took into consideration the frequency of the data. For example, if an idea or concept from a single participant was incongruent with the ideas/concepts provided by several other participants, competency statement rewrite followed the idea/concept with appeared with greater frequency for round 2 consideration. Throughout the data analysis process, CP maintained an audit trail of coding decisions and had regular meetings with CB where decisions were challenged to enhance rigor of analysis.

**Fig. 2:**
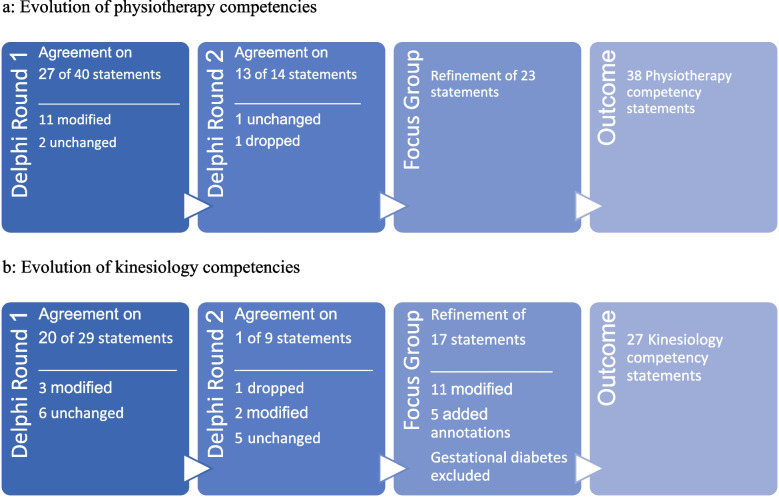
**a** Evolution of physiotherapy competencies. **b** Evolution of kinesiology competencies

The first Delphi round had a response rate of 81.8% (*n* = 9) for physiotherapists and 100% (*n* = 3) for kinesiologists. In the first round, 27 of the 40 physiotherapy competency statements and 20 of the 29 kinesiology statements achieved agreement consensus. Open text responses related to improving reading clarity of the statements or adding/removing specific skills from the statements. As a result of this feedback, 11 physiotherapy and three kinesiology competency statements were modified. The remaining two physiotherapy and six kinesiology statements that did not achieve consensus were left unchanged as there were either no comments to indicate why participants disagreed with the statement, or the comments were incongruent with their Likert scale rating. Qualitative feedback from physiotherapy participants indicated that one competency statement (which achieved agreement consensus) should be modified and another statement (which did not achieve consensus) did not reflect the intervention, population or practice setting of interest in this study. As a result of this feedback, the former was modified, and the latter returned to participants in round 2 with the suggestion to remove it from the final competency list. This resulted in a total of 14 physiotherapy competency statements for participants to consider in round 2. See Fig. 2 for competency statement evolution.

#### Delphi survey round 2

The round 2 surveys only presented the statements with remaining disagreement or those statements that were modified based on feedback from the first Delphi round (14 physiotherapy competencies, 9 kinesiology competencies) [See Additional files 3 and 4]. The surveys included feedback on the ratings from round 1 including the individual participant’s ratings for each competency statement, and the median and range of rating scores from all participants. This gave participants the opportunity to reconsider their opinion while remaining anonymous to other participants. Participants were asked to rate their level of agreement on the competencies included in the round 2 survey using the same five-points Likert scale as round 1, and were invited through open comment boxes, to provide feedback on why they might have changed or stayed their opinion.

Delphi round 2 had a 100% response rate for both groups (*n* = 11 for physiotherapists and *n* = 3 for kinesiologists). The analysis process used for Delphi round 1 was repeated for round 2. In round 2, there was agreement consensus from physiotherapy participants to drop the statement which did not reflect the intervention, population and practice setting of this study. All other physiotherapy competency statements achieved agreement consensus except for one. Only one kinesiology competency statement achieved agreement consensus and two were modified to reflect qualitative feedback. One kinesiology competency statement was dropped, even though only 33% disagreed with it, as we felt that the open-ended comments indicated that all participants did not feel this was a competency held by all entry-level CKA affiliated kinesiologists. For example, despite rating *neutral* on the Likert scale, open text comments were as follows from two separate participants, “Given that this area is not a required course [of those needed for affiliation with CKA] someone could not cover this area, i don’t agree that all Aff[illiated] Kin[esiologists] will have this competency” and, “After careful consideration, I feel this statement is neutral. An entry level Kin[esiologist] may not have in-depth knowledge in all these areas”. The remaining five kinesiology statements and one physiotherapy statement were left unmodified as qualitative data was either unclear or incongruent with participants' level of agreement ratings. No further survey rounds were pursued for competency statements remaining that did not achieve consensus the literature indicates that third Delphi rounds are unlikely to show significant increases in consensus [27] and may induce sample fatigue [29]. Instead, we ensured that these competencies were discussed in the focus groups.

#### Development of draft clinical tools for presentation to focus groups

The competency statements that were developed by subject matter experts in the two Delphi rounds describe the abilities of entry-level physiotherapists and kinesiologists in the specific clinical context of physical activity intervention delivery for diabetes care in primary care. These competencies were used to draft three clinical tools: 1) a physiotherapy competency profile, 2) a kinesiology competency profile and, 3) a referral pathway for physical activity interventions for diabetes care. The competency profiles were designed to provide role clarity for interprofessional primary care team members regarding the role of physiotherapists and kinesiologists on primary care teams for diabetes care. Each profile included the competency statements developed from the Delphi rounds with a preamble. The preamble included the purpose of the document and background information on each of the professions in relation to educational requirements and regulations.

The draft referral pathway was designed to translate the developed competencies into a clinical tool that supports appropriate referral generation to physiotherapists and kinesiologists delivering physical activity interventions for diabetes management in primary care. The referral pathway was developed with guidance from Stout et al. [39] who created an exercise referral clinical pathway for oncology. We used the health dimensions in Stout et al. work to create groups of factors that influence the risk and complexity of physical activity interventions for diabetes care: diabetes complications, medical considerations, mobility, and personal/environmental factors. Using these factors, this tool prompts primary care team members to consider the clinical profile of the person living with/at risk of diabetes and supports clinical decision making regarding physical activity referrals to exercise specialists.

#### Focus groups

Following the Delphi rounds, focus groups were scheduled to include as many participants from the Delphi rounds as possible. Eight physiotherapists (73% of Delphi participants) and three kinesiologists (100% of Delphi participants) participated in separate virtual focus groups. One kinesiology participant was unable to attend the focus group and alternatively, participated in a semi-structured interview to ensure we captured as much data as possible. The purpose of the focus groups was to elicit feedback on: a) how well the competency statements developed through the Delphi rounds represent the competencies of entry-level exercise specialists related to physical activity interventions for diabetes care in primary care (member-checking) and b) how well the referral pathway tool represents those competencies (face validity). Focus groups were chosen to allow for the free flow of information and ideas from the expert panel [28, 37].

Participants were sent a copy of their discipline specific draft competency profile and the draft referral pathway one week prior to the focus groups. Focus groups took place over video conferencing and were facilitated by CP with support from CB using a semi-structured interview guide which was developed for this study [See Additional file 5]. Audio–video recordings of the focus groups were transcribed using Microsoft Teams and were verified for accuracy by the primary investigator. Focus group transcripts were analyzed using content analysis [38] using qualitative data analysis software NVivo to organize and categorize the data [40], and the same analytic process described above in the Delphi survey round 1 section. The findings informed modifications to the draft competency profiles and the draft referral pathway and to determine face validity and usefulness of the clinical tools.

Focus group feedback regarding the competency statements resulted in changes to 23 physiotherapy statements and 17 kinesiology statements. While the nature of responses from kinesiology participants were similar across the focus group and semi-structured interview formats, feedback from the focus group was more comprehensive as participants had the opportunity to hear different perspectives. The physiotherapy focus group discussion identified specific skills that were missing from the competency profile, clarified wording to more accurately reflect the skills and abilities of entry-level physiotherapists and identified minor errors like redundancies. Kinesiologist participant feedback also identified wording changes that would more accurately reflect the skills and abilities of entry-level kinesiologists. The most significant findings, however, were related to the limits of practice for kinesiologists. This included that entry-level kinesiologists require additional training and mentorship for:

- managing hypoglycemia, pseudo-hypoglycemia, and acute hyperglycemia for physical activity intervention for people living with all types of diabetes,
- implementation and monitoring of physical activity in people living with type 1 diabetes, and
- supporting physical activity interventions in pregnancy.

Feedback about the draft referral pathway tool from physiotherapy and kinesiology participants identified ways to make the tool more readable and to incorporate the need for additional mentorship and training of kinesiologists with certain client populations. Otherwise, both physiotherapists and kinesiologists felt the referral pathway had good face validity and accurately reflected the competencies of their respective entry-level clinicians. Participants felt the developed tools could be useful for exercise specialists to understand their own role, understand each other’s roles and communicate those roles effectively to employers and other primary care team members.

## Results

Findings from the three stages of data collection and analysis in this study resulted in subject matter expert agreement on 38 physiotherapy competency statements and 27 kinesiology competency statements related to physical activity intervention for diabetes care in primary care settings. The final versions of the competency profiles are in Additional file 6. Fig. 1 and Additional file 7. Fig. 2.

**Fig. 3:**
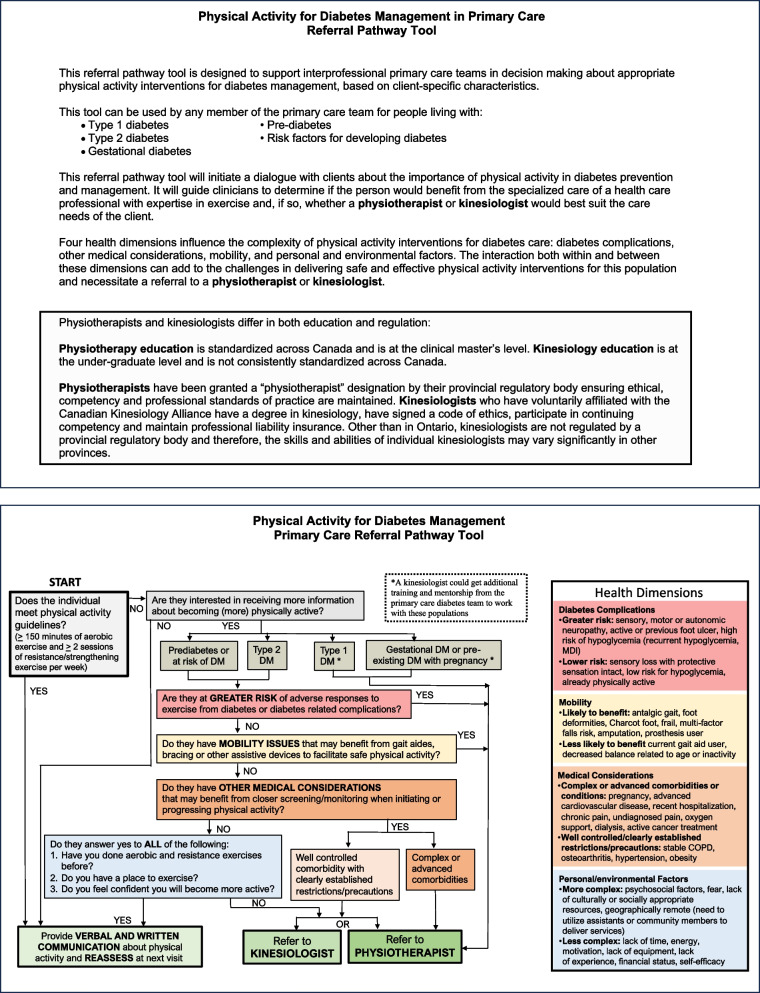
Physical activity referral pathway tool

The final version of the referral pathway is in Fig. 3. The clinical decision tool represents how a physical activity intervention referral will depend on the clinical profile of the person at risk for/living with diabetes. Both exercise specialists have the knowledge and skills to support people living with diabetes who have medical, physical, or personal or environmental barriers to becoming (more) physically active, and physiotherapists can support people whose medical or physical needs are complex.

## Discussion

The purpose of this study was to establish the competencies of two exercise specialist professions for delivering physical activity intervention for diabetes management in primary care settings, and using those competencies, to develop a referral pathway tool to differentiate the roles of physiotherapists and kinesiologists. This study aimed to fill a gap in the literature regarding the role of exercise specialists in diabetes management in primary care to improve primary care team interprofessional collaboration.

### Importance of professional specificity

The physiotherapy competency profile builds upon the existing national physiotherapy competency profile [33] by outlining the specific competencies that physiotherapists bring to physical activity interventions for diabetes management in primary care, adding context specificity which did not previously exist. The kinesiology competency profile, to our knowledge, is the first evidence informed national competency profile for kinesiologists related to diabetes care. The development of these two competency profiles in parallel demonstrated that there are differences in the roles of entry-level kinesiologists and physiotherapists with physical activity intervention delivery for diabetes management in primary care settings. Many physical activity intervention studies do not specify the profession of the person delivering physical activity interventions [5, 19], using generic terms, such as exercise specialists. Our study shows that professional specificity is important because while there are similarities between physiotherapists and kinesiologists, there are also differences in their professional competencies and the type of care they can provide to people living with diabetes. The developed referral pathway tool from this study is a unique clinical contribution to primary care teams by using client populations and characteristics to convey the distinct roles of physiotherapists and kinesiologists to promote appropriate referrals for physical activity interventions.

### Role of physiotherapists in primary care

This study has provided more specificity to the already existing body of literature on the role of physiotherapists in primary care. Other work has focused on primary care physiotherapists’ efficacy and contributions in more traditional physiotherapy roles such as management of musculoskeletal conditions [41–43]. Studies have found there is work to be done in educating providers (physicians and nurse practitioners) about the role of physiotherapists in primary care [44, 45], including their role in diabetes care. Janssen et al. [44] found that physiotherapists thought prevention and management of type 2 diabetes was well within their scope of practice, but they described barriers to providing this care. Barriers included a poor understanding of physicians about the role of physiotherapists in diabetes care, and poor care access due to lengthy waitlists in publicly funded practice settings. The impact of these barriers was that physiotherapists were missing the opportunity to provide care before the complications of diabetes had a significant impact on an individual’s overall function [44].

### Role of kinesiologists in primary care

For the profession of kinesiology, this study is the first step in establishing the role of kinesiologists in primary care, as we know of no other literature addressing this area other than commentary articles [46, 47] and exercise intervention studies [48–50]. While this study was an important first step in defining the role of kinesiology in physical activity intervention delivery for diabetes care in primary care, further research to validate these tools using a broader and larger sample from across Canada would be an important next step. The need for further validation of kinesiology competencies is especially true considering that kinesiologist education is not standardized across Canada the way other health care professional education is [51]. As a result, kinesiologists from different institutions may graduate with different competencies. Further, some kinesiologists obtain additional certifications from the Canadian Society for Exercise Physiology (CSEP) that may expand or change their competencies in diabetes care. A limitation of this study is that the developed tools may over- or under-represent the skills and abilities of some entry-level kinesiologists because of this educational inconsistency.

### Vital members of interprofessional diabetes care team

This study adds to the research literature by providing evidence on the role of physiotherapists and kinesiologists in physical activity interventions for diabetes care. This topic has been lacking in the literature to date, which is highlighted by their absence in the Clinical Practice Guidelines (CPG) [31]. Diabetes Canada’s CPGs are the gold standard for diabetes management in primary care settings, providing practice direction to a diverse group of health care providers [52]. In these CPGs there is guidance on referrals to other professionals such as dietitians [53]. However, there are no clear recommendations on making referrals to an exercise specialist for physical activity intervention support [7]. Based on the findings from this study, the CPGs would also benefit from including specific recommendations for referrals to exercise specialists like physiotherapists and kinesiologists to support these complex interventions. Further, the findings of this study suggest that the guidelines should provide guidance on the credentials of various types of exercise specialists since they have differing competencies.

There is no debate that exercise is effective for diabetes prevention, management, and complication prevention, however activity levels of Canadians indicate it is underutilized [1]. Recent literature to address this issue in primary care has focused on how to build capacity of existing clinicians like physicians and nurse practitioners [11, 54]. The findings of our study support the use of physiotherapists and kinesiologists to support primary care providers who have expressed challenges in practice like limited knowledge, time, and confidence in their ability to provide exercise interventions effectively, especially for those using insulin or with comorbidities [10, 11, 17, 55]. Beyond supporting effective referral generation, these competency profiles and referral pathway tool can support policymakers and interprofessional team decision-making about the inclusion of exercise specialists on primary care teams.

### Strengths

The first strength of this project was the quality of participants and their engagement in the research study. Participants were a heterogeneous group of clinicians with experience in primary care and specialty clinics, and academic researchers with expertise in diabetes. With representation from four provinces from east to west including participants who work with remote, northern, and Indigenous communities, participants brought a variety of perspectives to this study. Additionally with high response rates, participants were highly engaged throughout all stages.

The second strength of this study was the steps taken to ensure trustworthiness of our findings. The data triangulation of quantitative and qualitative data through repeated rounds and member-checking of the findings increase confidence that the results accurately reflect the opinion of subject matter experts. Since most competencies had achieved consensus through two Delphi survey rounds, we anticipated little modifications would be needed to the clinical tools at the focus group stage. However, focus groups discussions resulted in modifications to 40 competency statements including 33 of the 59 statements that had achieved consensus. The inclusion of focus groups following the survey rounds, captured both big and small concepts that were not seen in the survey rounds. For kinesiologist participants, focus group discussion clarified that certain competency statements were appropriate when working with people living with prediabetes or type 2 diabetes, but certain clinical skills needed for people living with type 1 diabetes or gestational diabetes would be beyond entry-level clinicians’ competencies. The robust interactive discussion in the focus group facilitated the development of an idea to add annotations as some mentorship and training would be sufficient to build those competencies. For physiotherapist participants, the changes were not as significant as that of the kinesiologists, but they were invested in ensuring the wording of each competency statement was accurate. For example, the inclusion of family and community in education and intervention competencies. This result reaffirms what some authors have said previously, which is that Delphi survey results should be further refined through additional research methods [28]. Bringing experts together from diverse practice settings in the focus groups allowed for refinement of ideas and increased clarity and confidence in the Delphi results from this study.

### Limitations

The first limitation of this study was the limited number of kinesiology participants. Although the sample was diverse, three participants may not be enough to represent the diversity of kinesiology education across Canada. Additionally, one of those participants was not able to attend the focus group and instead participated in a semi-structured interview and did not have the opportunity to hear other perspectives.

Another limitation is that the competency profiles and the referral pathway tool are for entry-level clinicians and do not account for additional education/training that physiotherapists or kinesiologists may bring with them to a primary care team. For example, regulated kinesiologists in Ontario and physiotherapists across Canada who have practical diabetes experience can apply to the Canadian Diabetes Educator Certification Board (CDECB) to become Certified Diabetes Educators (CDE)® [34]. Kinesiologists with practical experience and specific core competencies can apply to the Canadian Society for Exercise Physiology (CSEP) to become Clinical Exercise Physiologists™ [56]. Future research in this area may work to differentiate the competencies of entry-level physiotherapists and kinesiologists from these subsets of exercise professionals such as CSEP Clinical Exercise Physiologists™, regulated kinesiologists in Ontario or CDECB Certified Diabetes Educators (CDE)®. This would further support role clarity for exercise professionals and other members of primary care teams.

Another limitation of this study is that participants did not give feedback on the final versions of the clinical tools. Following the focus group stage, changes were made to the competency profiles and the referral pathway tool to address participants’ feedback and improve the tools. Further member-checking would increase confidence in the validity of the final version of the clinical tools. Additionally, the usefulness of the referral pathway tool should be evaluated by other members of the primary care interprofessional team.

## Conclusions

This study provides a unique contribution to research and clinical care for the prevention and management of diabetes using physical activity interventions. This study was the first to establish the specific competencies of physiotherapists and kinesiologists in diabetes care in primary care and the first to explore the differences between these professions. The findings highlight that physical activity interventions can be complex and that both exercise specialist professions have fundamental skills and abilities to support people living with diabetes to engage in physical activity. However, the findings also underscore that physiotherapy and kinesiology are unique health professions with entry-level clinicians possessing distinct skills and abilities which influence the type of care they can provide in primary care settings. This study highlights the importance of physiotherapists and kinesiologists as vital members of primary care teams for diabetes care, but also, the need for specificity when referring to “exercise specialists” in clinical practice guidelines, amongst primary care teams and in future research related to physical activity interventions for diabetes care.

## Supplementary Information

Additional file 1. Delphi survey. Round 1 (physiotherapy).

Additional file 2. Delphi survey. Round 1 (kinesiology).

Additional file 3. Delphi survey. Round 2 (physiotherapy).

Additional file 4. Delphi survey. Round 2 (kinesiology).

Additional file 5. Focus group interview guide. Semi-structure interview discussion guide (physiotherapy and kinesiology).

Additional file 6. Fig. 1: Physiotherapy competency profile.

Additional file 7. Fig. 2: Kinesiology competency profile.

## Data Availability

The datasets used and analyzed during the current study are available at an aggregated level to protect participant confidentiality from the corresponding author on reasonable request.

## Abbreviations

PARS: Physical Activity Referral Schemes
ERS: Exercise Referral Schemes
CDE: Certified Diabetes Educator®
NPAG: National Physiotherapy Advisory Group
CPG: Clinical Practice Guidelines
REDCap: Research Electronic Data Capture
CSEP: Canadian Society for Exercise Physiology
