# Supplementary material for: Referral pathway and competency profiles of primary care physiotherapists and kinesiologists for physical activity interventions for diabetes: a modified Delphi study
Source: BMC Prim Care. 2024 Oct 15;25:368. doi: 10.1186/s12875-024-02611-1 (PMC11479570; doi:10.1186/s12875-024-02611-1)
Supplement: Supplementary file 1 — Additional file 1. Delphi survey. Round 1 (physiotherapy). [file 12875_2024_2611_MOESM1_ESM.docx]

**Physiotherapy Delphi Round One Survey**

The purpose of this survey is to **establish the common and distinct competencies of entry-level physiotherapists and kinesiologists** in physical activity for diabetes in primary care settings. The results will be used to develop a referral pathway tool for diabetes care by exercise specialists, for use by interprofessional primary care teams.

The [*Competency Profile for Physiotherapists in Canada*](http://chrome-extension/efaidnbmnnnibpcajpcglclefindmkaj/viewer.html?pdfurl=https%3A%2F%2Fwww.peac-aepc.ca%2Fpdfs%2FResources%2FCompetency%2520Profiles%2FCompetency%2520Profile%2520for%2520PTs%25202017%2520EN.pdf&clen=413214&chunk=true) outlines the essential competencies of entry-level physiotherapists. These essential competencies are the “required ability of a physiotherapist” at the point of entry-to-practice^1^.

For this survey, competency statements that were relevant to physical activity interventions for diabetes care in primary care settings were modified to reflect the intervention, patient population and clinical context. The modified competencies primarily fell into 4 of the 7 domains: Physiotherapy expertise, collaboration, scholarship, and professionalism.

The following section of the survey consists of 40 proposed competency statements for **entry-level physiotherapists related to physical activity intervention for diabetes care in primary care settings**.

You will be asked to **rate your level of agreement** with each competency statement. If applicable, include comments or suggestions about how you would modify the statement to better reflect the competencies held by **all entry-level physiotherapists**. You could either rephrase the statement or tell us what should be added, what should be removed or what should be changed. For example, in a competency statement about treatment modalities, you may agree with most of the statement, but think that not all entry-level physiotherapists would be proficient with one of the modalities mentioned. In the comments section you could write “*remove XX modality*” or you could rewrite the statement yourself with the modality removed. If you feel a competency statement should be added, please include it in your comments.

1. National Physiotherapy Advisory Group. (2017). *Competency profile for physiotherapist in Canada*. <https://www.peac-aepc.ca/pdfs/Resources/Competency%20Profiles/Competency%20Profile%20for%20PTs%202017%20EN.pdf>

____________________________________________________________________

Domain: PHYSIOTHERAPY EXPERTISE

*Ensures physical and emotional safety of client*

Please **rate your level of agreement** with the following competency statements related to the **skills and** **abilities held by ALL licensed entry-level physiotherapists.**

If applicable, include comments or suggestions about how you would modify the statement.

1. Identifies client-specific precautions, contraindications and risks to physical activity participation from acute hyperglycemia, hypoglycemia or pseudo-hypoglycemia

[] [] [] [] []

Strongly agree Agree Neutral Disagree Strongly disagree

Comments: [if selected strongly agree]

Please explain your selection: [if selected any other response]

1. Identifies client-specific precautions, contraindications and risks to physical activity participation from preproliferative, proliferative retinopathy, autonomic neurological dysfunction, foot ulcer or pregnancy related complications in women with gestational diabetes

[] [] [] [] []

Strongly agree Agree Neutral Disagree Strongly disagree

Comments: [if selected strongly agree]

Please explain your selection: [if selected any other response]

1. Identifies client-specific precautions, contraindications and risks to physical activity participation from non-diabetes related comorbidities in people living with diabetes

[] [] [] [] []

Strongly agree Agree Neutral Disagree Strongly disagree

Comments: [if selected strongly agree]

Please explain your selection: [if selected any other response]

1. Monitors relevant parameters including blood pressure, oximetry, heart rate, and respiratory rate during assessment and physical activity intervention that enhances the client's safety and comfort

[] [] [] [] []

Strongly agree Agree Neutral Disagree Strongly disagree

Comments: [if selected strongly agree]

Please explain your selection: [if selected any other response]

1. Identifies need for and makes recommendations for ambulatory, assistive, adaptive, and protective devices to support client's safety and comfort when participating in physical activity

*(Ambulatory devices: canes, crutches, walkers, walking poles, wheelchairs; Assistive, adaptive, protective devices: splints, taping/wrapping, bandaging, braces, orthotics, garments, collars)^1^*

[] [] [] [] []

Strongly agree Agree Neutral Disagree Strongly disagree

Comments: [if selected strongly agree]

Please explain your selection: [if selected any other response]

1. Performs foot assessment including peripheral circulation, sensory testing and skin integrity to ensure client's safety and comfort when participating in physical activity

[] [] [] [] []

Strongly agree Agree Neutral Disagree Strongly disagree

Comments: [if selected strongly agree]

Please explain your selection: [if selected any other response]

1. Identifies signs and symptoms of hypoglycemic and hyperglycemic emergencies in response to physical activity and takes appropriate action

[] [] [] [] []

Strongly agree Agree Neutral Disagree Strongly disagree

Comments: [if selected strongly agree]

Please explain your selection: [if selected any other response]

1. Identifies and responds to non-glycemic adverse responses to physical activity interventions for diabetes management with exercise modifications, education and/or consultation with appropriate health care provider

[] [] [] [] []

Strongly agree Agree Neutral Disagree Strongly disagree

Comments: [if selected strongly agree]

Please explain your selection: [if selected any other response]

___________________________________________________________________

Domain: PHYSIOTHERAPY EXPERTISE (continued)

*Conducts client assessment*

Please **rate your level of agreement** with the following competency statements related to the **skills and** **abilities held by ALL licensed entry-level physiotherapists.**

If applicable, include comments or suggestions about how you would modify the statement.

1. Interviews clients living with type 1, type 2 or gestational diabetes to obtain relevant information about diabetes, other health conditions, and personal and environmental factors relevant to physical activity for diabetes management

[] [] [] [] []

Strongly agree Agree Neutral Disagree Strongly disagree

Comments: [if selected strongly agree]

Please explain your selection: [if selected any other response]

1. Interviews clients to determine their knowledge of diabetes, current self-management skills, and stage of behavior change and adjusts assessment and treatment plan accordingly

[] [] [] [] []

Strongly agree Agree Neutral Disagree Strongly disagree

Comments: [if selected strongly agree]

Please explain your selection: [if selected any other response]

1. Obtains information about client's status from lab work, diagnostic imaging, electrocardiogram or pulmonary function test results relevant to physical activity for diabetes management

[] [] [] [] []

Strongly agree Agree Neutral Disagree Strongly disagree

Comments: [if selected strongly agree]

Please explain your selection: [if selected any other response]

1. Identifies risk factors such as comorbidities, smoking, nutritional, alcohol/drug use, and activity level that place healthy or pre-diabetes populations at high risk for developing diabetes and those already living with diabetes, at high risk of developing diabetes related complications

[] [] [] [] []

Strongly agree Agree Neutral Disagree Strongly disagree

Comments: [if selected strongly agree]

Please explain your selection: [if selected any other response]

1. As a primary care practitioner, identifies yellow, orange or red flags and makes appropriate referrals to other team members and/or makes appropriate changes to assessment and treatment plans for physical activity interventions for diabetes care *(Red flags: signs of serious pathology; Orange flags: psychiatric symptoms; Yellow flags: maladaptive pain coping strategies) ^2^*

[] [] [] [] []

Strongly agree Agree Neutral Disagree Strongly disagree

Comments: [if selected strongly agree]

Please explain your selection: [if selected any other response]

1. Identifies wounds that may benefit from referral to other services, and advises clients accordingly

[] [] [] [] []

Strongly agree Agree Neutral Disagree Strongly disagree

Comments: [if selected strongly agree]

Please explain your selection: [if selected any other response]

1. Identifies inappropriately fitting prothesis that may benefit from referral to other services, and advises clients accordingly

[] [] [] [] []

Strongly agree Agree Neutral Disagree Strongly disagree

Comments: [if selected strongly agree]

Please explain your selection: [if selected any other response]

1. Selects and performs appropriate tests and measures to identify current fitness level and potential barriers to physical activity for diabetes management including pelvic ligament laxity, diastasis recti, impaired skin integrity, respiratory, vascular or neurological impairments, cognitive or mental health disorders, musculoskeletal injury or chronic pain

[] [] [] [] []

Strongly agree Agree Neutral Disagree Strongly disagree

Comments: [if selected strongly agree]

Please explain your selection: [if selected any other response]

1. Interprets assessment findings and develops a physiotherapy diagnosis and working prognosis in order to design appropriate physical activity intervention for diabetes management

(*Physiotherapy diagnosis: A conclusion about physical function based on a subjective and objective assessment and analysis by a physiotherapist to investigate the cause or nature of a client’s condition or problem)^1^*

[] [] [] [] []

Strongly agree Agree Neutral Disagree Strongly disagree

Comments: [if selected strongly agree]

Please explain your selection: [if selected any other response]

_____________________________________________________________________

Domain: PHYSIOTHERAPY EXPERTISE (continued)

*Develops, implements, monitors and evaluates an intervention plan*

Please **rate your level of agreement** with the following competency statements related to the **skills and** **abilities held by ALL licensed entry-level physiotherapists.**

If applicable, include comments or suggestions about how you would modify the statement.

1. Establishes a physical activity goal for diabetes management in collaboration with client and develops an intervention plan appropriate to the client’s goals, current stage of change, current health status and personal and environmental factors

[] [] [] [] []

Strongly agree Agree Neutral Disagree Strongly disagree

Comments: [if selected strongly agree]

Please explain your selection: [if selected any other response]

1. Educates clients living with type 1, type 2 and gestational diabetes about the benefits and risks of various physical activities with consideration for individual’s existing comorbidities

[] [] [] [] []

Strongly agree Agree Neutral Disagree Strongly disagree

Comments: [if selected strongly agree]

Please explain your selection: [if selected any other response]

1. Implements and monitors customized physical activity intervention including aerobic, aquatic, strength, flexibility and/or balance exercises designed to optimize glycemic control, cardiorespiratory fitness, diabetes complication risk and/or quality of life

[] [] [] [] []

Strongly agree Agree Neutral Disagree Strongly disagree

Comments: [if selected strongly agree]

Please explain your selection: [if selected any other response]

1. Assists clients to develop self-management skills in physical activity, foot care and nutrition counselling to support physical activity for diabetes management

[] [] [] [] []

Strongly agree Agree Neutral Disagree Strongly disagree

Comments: [if selected strongly agree]

Please explain your selection: [if selected any other response]

1. Identifies strategies to manage the hypoglycemic effect of physical activity for clients who use insulin or hyperglycemic medications with a risk of hypoglycemia

[] [] [] [] []

Strongly agree Agree Neutral Disagree Strongly disagree

Comments: [if selected strongly agree]

Please explain your selection: [if selected any other response]

1. Supports clients with comorbidities to perform physical activity for diabetes management through therapeutic interventions including oxygen titration, breathing strategies, energy conservation, pelvic floor exercises, wound care, sensory training/retraining, electrophysical agents, spinal/peripheral joint mobilizations, neurodynamic techniques, and wheelchair skills

[] [] [] [] []

Strongly agree Agree Neutral Disagree Strongly disagree

Comments: [if selected strongly agree]

Please explain your selection: [if selected any other response]

1. Monitors skin, wounds, blisters and scars during physical activity for diabetes management and responds appropriately with basic wound hygiene and self-management education

[] [] [] [] []

Strongly agree Agree Neutral Disagree Strongly disagree

Comments: [if selected strongly agree]

Please explain your selection: [if selected any other response]

1. Monitors client’s response to physical activity intervention for diabetes management, reassesses client's needs and modifies physical activity intervention plan as indicated

[] [] [] [] []

Strongly agree Agree Neutral Disagree Strongly disagree

Comments: [if selected strongly agree]

Please explain your selection: [if selected any other response]

1. Identifies opportunities for group physical activity programming for diabetes management

[] [] [] [] []

Strongly agree Agree Neutral Disagree Strongly disagree

Comments: [if selected strongly agree]

Please explain your selection: [if selected any other response]

1. Plans, delivers and evaluates group physical activity programming for diabetes management

[] [] [] [] []

Strongly agree Agree Neutral Disagree Strongly disagree

Comments: [if selected strongly agree]

Please explain your selection: [if selected any other response]

_____________________________________________________________________

Domain: COLLABORATION

Please **rate your level of agreement** with the following competency statements related to the **skills and** **abilities held by ALL licensed entry-level physiotherapists.**

If applicable, include comments or suggestions about how you would modify the statement.

1. Employs a client-centered approach by acting in a manner that respects client uniqueness, diversity and autonomy, with the client as a key member of the diabetes management care team

*(Diversity: refers to variation among people including, but not limited to, variation based upon factors such as race, ethnicity, colour, religion, age, sex, sexual orientation, marital status, family status, and disability)^1^*

[] [] [] [] []

Strongly agree Agree Neutral Disagree Strongly disagree

Comments: [if selected strongly agree]

Please explain your selection: [if selected any other response]

1. Facilitates collaborative relationships with interprofessional diabetes care team

[] [] [] [] []

Strongly agree Agree Neutral Disagree Strongly disagree

Comments: [if selected strongly agree]

Please explain your selection: [if selected any other response]

1. Provides services that balance waitlists, client needs and available resources including delegating care to and supervising personnel involved in physiotherapy service delivery for diabetes care

*(Personnel involved in physiotherapy service delivery: includes support personnel, assistants, volunteers, and other healthcare providers, who may provide physiotherapy services under the direction and supervision of a physiotherapist)^1^*

[] [] [] [] []

Strongly agree Agree Neutral Disagree Strongly disagree

Comments: [if selected strongly agree]

Please explain your selection: [if selected any other response]

1. Identifies the learning needs related to physical activity and diabetes management of other healthcare providers and contributes to and assesses the effectiveness of learning activities

[] [] [] [] []

Strongly agree Agree Neutral Disagree Strongly disagree

Comments: [if selected strongly agree]

Please explain your selection: [if selected any other response]

_________________________________________________________________

Domain: SCHOLARSHIP

Please **rate your level of agreement** with the following competency statements related to the **skills and** **abilities held by ALL licensed entry-level physiotherapists.**

If applicable, include comments or suggestions about how you would modify the statement.

1. Able to access and critically appraise emerging information relevant to physical activity and diabetes management and determine potential for applicability in primary care settings

[] [] [] [] []

Strongly agree Agree Neutral Disagree Strongly disagree

Comments: [if selected strongly agree]

Please explain your selection: [if selected any other response]

1. Uses a structured evidence-informed approach incorporating best available evidence, client context and personal knowledge and experience into clinical decision making for diabetes care

[] [] [] [] []

Strongly agree Agree Neutral Disagree Strongly disagree

Comments: [if selected strongly agree]

Please explain your selection: [if selected any other response]

__________________________________________________________________

Domain: PROFESSIONALISM

Please **rate your level of agreement** with the following competency statements related to the **skills and** **abilities held by ALL licensed entry-level physiotherapists.**

If applicable, include comments or suggestions about how you would modify the statement.

1. Advocates for the value of physiotherapy services to overcome barriers and facilitate improved physical activity interventions in diabetes care in primary care settings

*(Physiotherapy services: services provided by or under the direction of a physiotherapist. This includes client assessment and intervention, and related communication with and reporting to various parties for the purposes of delivering client care)^1^*

[] [] [] [] []

Strongly agree Agree Neutral Disagree Strongly disagree

Comments: [if selected strongly agree]

Please explain your selection: [if selected any other response]

1. Has been granted a “physiotherapist” designation by their provincial regulatory body which, through delegated authority from the provincial government, ensures ethical, competency and professional standards of practice are maintained using mechanisms including a public registry of individual physiotherapists and a robust complaint, investigative and disciplinary program with input from members of the public

[] [] [] [] []

Strongly agree Agree Neutral Disagree Strongly disagree

Comments: [if selected strongly agree]

Please explain your selection: [if selected any other response]

1. Recognizes and addresses real, potential or perceived conflicts of interest with pharmaceutical companies and fitness facilities/vendors

[] [] [] [] []

Strongly agree Agree Neutral Disagree Strongly disagree

Comments: [if selected strongly agree]

Please explain your selection: [if selected any other response]

1. Demonstrates awareness of the social determinants of health in diabetes management and advocates for physical activity opportunities and support that are sustainable, socially, and culturally appropriate and geographically accessible to clients year round

[[] [] [] [] []

Strongly agree Agree Neutral Disagree Strongly disagree

Comments: [if selected strongly agree]

Please explain your selection: [if selected any other response]

1. Recognizes, explores and acknowledges the relationship between the legacy of colonization and current high rates of diabetes amongst Indigenous peoples

[] [] [] [] []

Strongly agree Agree Neutral Disagree Strongly disagree

Comments: [if selected strongly agree]

Please explain your selection: [if selected any other response]

1. Incorporates a purposeful process of learning and continuous self-reflection recognizing one’s own concepts of health, diabetes care and assumptions about Indigenous peoples and respecting an Indigenous person’s preferences and barriers to re-connecting and integrating cultural resources and traditional approaches to care

[] [] [] [] []

Strongly agree Agree Neutral Disagree Strongly disagree

Comments: [if selected strongly agree]

Please explain your selection: [if selected any other response]

1. Understands and works within physiotherapy scope of practice and personal level of competence in diabetes management as required by licensing body

[] [] [] [] []

Strongly agree Agree Neutral Disagree Strongly disagree

Comments: [if selected strongly agree]

Please explain your selection: [if selected any other response]

Three practice domains from the [*Competency Profile for Physiotherapists in Canada*](http://chrome-extension/efaidnbmnnnibpcajpcglclefindmkaj/viewer.html?pdfurl=https%3A%2F%2Fwww.peac-aepc.ca%2Fpdfs%2FResources%2FCompetency%2520Profiles%2FCompetency%2520Profile%2520for%2520PTs%25202017%2520EN.pdf&clen=413214&chunk=true) were not included in this survey (communication, management, and leadership). The competencies from these domains were either deemed to be not specifically relevant to physical activity interventions for diabetes care in primary care settings or were accounted for within the included domains in other competency statements.

Were there any competencies missing from this survey that you think should have been included related to communication, management, or leadership? If so please specify:

_________________________________________________________________________________________________________________________________________________________________________________________________________________

1. National Physiotherapy Advisory Group. (2017). *Competency profile for physiotherapist in Canada*. <https://www.peac-aepc.ca/pdfs/Resources/Competency%20Profiles/Competency%20Profile%20for%20PTs%202017%20EN.pdf>
2. Nicholas, M. K., Linton, S. J., Watson, P. J., & Main, C. J. (2011). Early identification and management of psychological risk factors (“yellow flags”) in patients with low back pain: A reappraisal. *Physical Therapy*, *91*(5), 737–753. <https://doi.org/10.2522/PTJ.20100224>

**This is the end of the survey.**

Once you click submit, you will not be able to return to the survey to make any further changes to your answers.

**Thank you for taking the time to complete the survey and for your collaboration in this research project!**
