# Supplementary material for: Referral pathway and competency profiles of primary care physiotherapists and kinesiologists for physical activity interventions for diabetes: a modified Delphi study
Source: BMC Prim Care. 2024 Oct 15;25:368. doi: 10.1186/s12875-024-02611-1 (PMC11479570; doi:10.1186/s12875-024-02611-1)
Supplement: Supplementary file 2 — Additional file 2. Delphi survey. Round 1 (kinesiology). [file 12875_2024_2611_MOESM2_ESM.docx]

**Kinesiology Delphi Round One Survey**

The purpose of this survey is to **establish the common and distinct competencies of entry-level physiotherapists and kinesiologists** in physical activity for diabetes in primary care settings. The results will be used to develop a referral pathway tool for diabetes care by exercise specialists, for use by interprofessional primary care teams.

The [*Competency Profile for Physiotherapists in Canada*](http://chrome-extension/efaidnbmnnnibpcajpcglclefindmkaj/viewer.html?pdfurl=https%3A%2F%2Fwww.peac-aepc.ca%2Fpdfs%2FResources%2FCompetency%2520Profiles%2FCompetency%2520Profile%2520for%2520PTs%25202017%2520EN.pdf&clen=413214&chunk=true) outlines the essential competencies of entry-level physiotherapists. These essential competencies are the “required ability of a physiotherapist” at the point of entry-to-practice^1^. The [*Canadian Kinesiology Alliance (CKA) Competency Profile*](https://www.cka.ca/en/competency-profile-competency-required) outlines the "knowledge and skills an entry-level practitioner should possess" at the point of entry-to-practice^2^.

For this survey, the *Competency Profile for Physiotherapists in Canada* was used as a framework and competency statements that were relevant to physical activity interventions for diabetes care in primary care settings were modified to reflect the intervention, patient population, clinical context and exercise professional (based on the CKA competency profile). The modified competencies primarily fell into 4 of the 7 domains: Kinesiology expertise, collaboration, scholarship, and professionalism.

The following section of the survey consists of 29 proposed competency statements for **entry-level kinesiologists (who are affiliated with the CKA) related to physical activity intervention for diabetes care in primary care settings**.

You will be asked to **rate your level of agreement** with each competency statement. If applicable, include comments or suggestions about how you would modify the statement to better reflect the competencies held by **all entry-level CKA affiliated kinesiologists**. You could either rephrase the statement or tell us what should be added, what should be removed or what should be changed. For example, in a competency statement about treatment modalities, you may agree with most of the statement but think that not all entry-level kinesiologists would be proficient with one of the modalities mentioned. In the comments section you could write “*remove XX modality*” or you could rewrite the statement yourself with the modality removed. If you feel a competency statement should be added, please include it in your comments.

1. National Physiotherapy Advisory Group. (2017). *Competency profile for physiotherapist in Canada*. <https://www.peac-aepc.ca/pdfs/Resources/Competency%20Profiles/Competency%20Profile%20for%20PTs%202017%20EN.pdf>

2. Canadian Kinesiology Alliance. (n.d.). *What are the requirements for affiliation?* Retrieved September 8, 2021, from <https://www.cka.ca/en/requirements-affiliation>

___________________________________________________________________

Domain: KINESIOLOGY EXPERTISE

*Ensures physical and emotional safety of client*

Please **rate your level of agreement** with the following competency statements related to the **skills and** **abilities held by ALL CKA affiliated entry-level kinesiologists.**

If applicable, include comments or suggestions about how you would modify the statement.

1. Identifies client-specific precautions, contraindications and risks to physical activity participation from acute hyperglycemia, hypoglycemia or pseudo-hypoglycemia

[] [] [] [] []

Strongly agree Agree Neutral Disagree Strongly disagree

Comments: [if selected strongly agree]

Please explain your selection: [if selected any other response]

1. Identifies client-specific precautions, contraindications and risks to physical activity participation from preproliferative, proliferative retinopathy, autonomic neurological dysfunction, foot ulcer or pregnancy related complications in women with gestational diabetes

[] [] [] [] []

Strongly agree Agree Neutral Disagree Strongly disagree

Comments: [if selected strongly agree]

Please explain your selection: [if selected any other response]

1. Identifies client-specific precautions, contraindications and risks to physical activity participation from non-diabetes related comorbidities in people living with diabetes

[] [] [] [] []

Strongly agree Agree Neutral Disagree Strongly disagree

Comments: [if selected strongly agree]

Please explain your selection: [if selected any other response]

1. Monitors relevant parameters including blood pressure, oximetry, heart rate, respiratory rate during assessment and physical activity interventions that enhances the client's safety and comfort

[] [] [] [] []

Strongly agree Agree Neutral Disagree Strongly disagree

Comments: [if selected strongly agree]

Please explain your selection: [if selected any other response]

1. Identifies signs and symptoms of hypoglycemic and hyperglycemic emergencies in response to physical activity and takes appropriate action

[] [] [] [] []

Strongly agree Agree Neutral Disagree Strongly disagree

Comments: [if selected strongly agree]

Please explain your selection: [if selected any other response]

1. Identifies and responds to non-glycemic adverse responses to physical activity interventions for diabetes management with exercise modifications, education and/or consultation with appropriate health care provider

[] [] [] [] []

Strongly agree Agree Neutral Disagree Strongly disagree

Comments: [if selected strongly agree]

Please explain your selection: [if selected any other response]

____________________________________________________________________

Domain: KINESIOLOGY EXPERTISE (continued)

*Conducts client assessment*

Please **rate your level of agreement** with the following competency statements related to the **skills and** **abilities held by ALL CKA affiliated entry-level kinesiologists.**

If applicable, include comments or suggestions about how you would modify the statement.

1. Interviews clients living with type 1, type 2 or gestational diabetes to obtain relevant information about diabetes, other health conditions, and personal and environmental factors relevant to physical activity for diabetes management

[] [] [] [] []

Strongly agree Agree Neutral Disagree Strongly disagree

Comments: [if selected strongly agree]

Please explain your selection: [if selected any other response]

1. Interviews client to determine their knowledge of diabetes, current self-management skills, and stage of behavior change and adjusts assessment and treatment plan accordingly

[] [] [] [] []

Strongly agree Agree Neutral Disagree Strongly disagree

Comments: [if selected strongly agree]

Please explain your selection: [if selected any other response]

1. Identifies risk factors such as comorbidities, smoking, nutritional, alcohol/drug use, and activity level that place healthy or pre-diabetes populations at high risk for developing diabetes and those already living with diabetes, at high risk of developing diabetes related complications.

[] [] [] [] []

Strongly agree Agree Neutral Disagree Strongly disagree

Comments: [if selected strongly agree]

Please explain your selection: [if selected any other response]

1. Selects and performs appropriate tests and measures to identify current fitness level and potential barriers to physical activity for diabetes management including pelvic ligament laxity, diastasis recti, impaired skin integrity, respiratory, vascular or neurological impairments, cognitive or mental health disorders, musculoskeletal injury or chronic pain

[] [] [] [] []

Strongly agree Agree Neutral Disagree Strongly disagree

Comments: [if selected strongly agree]

Please explain your selection: [if selected any other response]

1. Able to understand, evaluate, and interpret assessment findings and referral documentation to form a clinical impression

[] [] [] [] []

Strongly agree Agree Neutral Disagree Strongly disagree

Comments: [if selected strongly agree]

Please explain your selection: [if selected any other response]

_________________________________________________________________

Domain: KINESIOLOGY EXPERTISE (continued)

*Develops, implements, monitors and evaluates an intervention plan*

Please **rate your level of agreement** with the following competency statements related to the **skills and** **abilities held by ALL CKA affiliated entry-level kinesiologists.**

If applicable, include comments or suggestions about how you would modify the statement.

1. Develops an intervention plan appropriate to the client’s goals, current stage of change, current health status and personal and environmental factors

[] [] [] [] []

Strongly agree Agree Neutral Disagree Strongly disagree

Comments: [if selected strongly agree]

Please explain your selection: [if selected any other response]

1. Educates clients living with type 1, type 2 and gestational diabetes about the benefits of various physical activities

[] [] [] [] []

Strongly agree Agree Neutral Disagree Strongly disagree

Comments: [if selected strongly agree]

Please explain your selection: [if selected any other response]

1. Implements and monitors customized physical activity intervention including aerobic, aquatic, strength, flexibility and/or balance exercises designed to optimize glycemic control, cardiorespiratory fitness, diabetes complication risk and/or quality of life

[] [] [] [] []

Strongly agree Agree Neutral Disagree Strongly disagree

Comments: [if selected strongly agree]

Please explain your selection: [if selected any other response]

1. Assists clients to develop self-management skills in physical activity and nutrition counselling to support physical activity for diabetes management

[] [] [] [] []

Strongly agree Agree Neutral Disagree Strongly disagree

Comments: [if selected strongly agree]

Please explain your selection: [if selected any other response]

1. Identifies strategies to manage the hypoglycemic effect of physical activity for clients who use insulin or hyperglycemic medications with a risk of hypoglycemia

[] [] [] [] []

Strongly agree Agree Neutral Disagree Strongly disagree

Comments: [if selected strongly agree]

Please explain your selection: [if selected any other response]

1. Supports clients with comorbidities to perform physical activity for diabetes management through therapeutic interventions including ice, heat, exercise, taping, transcutaneous electrical nerve stimulation, and ultrasound.

[] [] [] [] []

Strongly agree Agree Neutral Disagree Strongly disagree

Comments: [if selected strongly agree]

Please explain your selection: [if selected any other response]

1. Monitors client’s response to physical activity intervention for diabetes management, reassesses client's needs and modifies physical activity intervention plan as indicated

[] [] [] [] []

Strongly agree Agree Neutral Disagree Strongly disagree

Comments: [if selected strongly agree]

Please explain your selection: [if selected any other response]

1. Identifies opportunities for group physical activity programming for diabetes management

[] [] [] [] []

Strongly agree Agree Neutral Disagree Strongly disagree

Comments: [if selected strongly agree]

Please explain your selection: [if selected any other response]

1. Plan, deliver and evaluate group physical activity programming for diabetes management

[] [] [] [] []

Strongly agree Agree Neutral Disagree Strongly disagree

Comments: [if selected strongly agree]

Please explain your selection: [if selected any other response]

_______________________________________________________________

Domain: COLLABORATION

Please **rate your level of agreement** with the following competency statements related to the **skills and** **abilities held by ALL CKA affiliated entry-level kinesiologists.**

If applicable, include comments or suggestions about how you would modify the statement.

1. Acts in a manner that respects clients’ diversity, autonomy and is in the best interest of the client when delivering physical activity interventions for diabetes management

*(Diversity: refers to variation among people including, but not limited to, variation based upon factors such as race, ethnicity, colour, religion, age, sex, sexual orientation, marital status, family status, and disability)^1^*

[] [] [] [] []

Strongly agree Agree Neutral Disagree Strongly disagree

Comments: [if selected strongly agree]

Please explain your selection: [if selected any other response]

1. Facilitates collaborative relationships with interprofessional diabetes care team

[] [] [] [] []

Strongly agree Agree Neutral Disagree Strongly disagree

Comments: [if selected strongly agree]

Please explain your selection: [if selected any other response]

_________________________________________________________________

Domain: SCHOLARSHIP

Please **rate your level of agreement** with the following competency statements related to the **skills and** **abilities held by ALL CKA affiliated entry-level kinesiologists.**

If applicable, include comments or suggestions about how you would modify the statement.

1. Able to use best practice guidelines including the interpretation and application of current evidence-based knowledge into clinical decision making for physical activity intervention in diabetes care

[] [] [] [] []

Strongly agree Agree Neutral Disagree Strongly disagree

Comments: [if selected strongly agree]

Please explain your selection: [if selected any other response]

________________________________________________________________

Domain: PROFESSIONALISM

Please **rate your level of agreement** with the following competency statements related to the **skills and** **abilities held by ALL CKA affiliated entry-level kinesiologists.**

If applicable, include comments or suggestions about how you would modify the statement.

1. Able to facilitate client access to kinesiology services and resources to physical activity interventions in diabetes care in primary care

[] [] [] [] []

Strongly agree Agree Neutral Disagree Strongly disagree

Comments: [if selected strongly agree]

Please explain your selection: [if selected any other response]

1. Complies with the code of ethics established by their professional body and is committed to continuing competency

[] [] [] [] []

Strongly agree Agree Neutral Disagree Strongly disagree

Comments: [if selected strongly agree]

Please explain your selection: [if selected any other response]

1. Recognizes and addresses conflicts of interest with pharmaceutical companies and fitness facilities/vendors

[] [] [] [] []

Strongly agree Agree Neutral Disagree Strongly disagree

Comments: [if selected strongly agree]

Please explain your selection: [if selected any other response]

1. Demonstrates awareness of the social determinants of health in diabetes management

[] [] [] [] []

Strongly agree Agree Neutral Disagree Strongly disagree

Comments: [if selected strongly agree]

Please explain your selection: [if selected any other response]

1. Recognizes, explores and acknowledges the relationship between the legacy of colonization and current high rates of diabetes amongst Indigenous peoples

[] [] [] [] []

Strongly agree Agree Neutral Disagree Strongly disagree

Comments: [if selected strongly agree]

Please explain your selection: [if selected any other response]

1. Understands and works within own professional knowledge, competence, and skill set in diabetes management

[] [] [] [] []

Strongly agree Agree Neutral Disagree Strongly disagree

Comments: [if selected strongly agree]

Please explain your selection: [if selected any other response]

Three practice domains from the [*Competency Profile for Physiotherapists in Canada*](http://chrome-extension/efaidnbmnnnibpcajpcglclefindmkaj/viewer.html?pdfurl=https%3A%2F%2Fwww.peac-aepc.ca%2Fpdfs%2FResources%2FCompetency%2520Profiles%2FCompetency%2520Profile%2520for%2520PTs%25202017%2520EN.pdf&clen=413214&chunk=true) were not included in this survey (communication, management, and leadership). The competencies from these domains were either deemed to be not specifically relevant to physical activity interventions for diabetes care in primary care settings or were accounted for within the included domains in other competency statements.

Were there any competencies missing from this survey that you think should have been included related to communication, management, or leadership? If so please specify:

_________________________________________________________________________________________________________________________________________________________________________________________________________________________________________

1. National Physiotherapy Advisory Group. (2017). *Competency profile for physiotherapist in Canada*. <https://www.peac-aepc.ca/pdfs/Resources/Competency%20Profiles/Competency%20Profile%20for%20PTs%202017%20EN.pdf>

**This is the end of the survey.**

Once you click submit, you will not be able to return to the survey to make any further changes to your answers.

**Thank you for taking the time to complete the survey and for your collaboration in this research project!**
