# Supplementary material for: Referral pathway and competency profiles of primary care physiotherapists and kinesiologists for physical activity interventions for diabetes: a modified Delphi study
Source: BMC Prim Care. 2024 Oct 15;25:368. doi: 10.1186/s12875-024-02611-1 (PMC11479570; doi:10.1186/s12875-024-02611-1)
Supplement: Supplementary file 3 — Additional file 3. Delphi survey. Round 2 (physiotherapy). [file 12875_2024_2611_MOESM3_ESM.docx]

**Physiotherapy Delphi Round Two Survey**

The following survey consists of 14 proposed competency statements for **entry-level physiotherapists related to physical activity interventions for diabetes care in primary care settings**.

Following Round 1, any competency statements that all or most of the expert physiotherapist panel agreed upon have been removed from the Round 2 survey. Those that did not achieve consensus and therefore need further review, have been modified based on your feedback and included in this survey.

To review, the purpose of this study is to understand both the similarities and the differences between the competencies of physiotherapists and kinesiologists in this practice area. The results will be used to develop a referral pathway tool for diabetes care by exercise specialists, for use by interprofessional primary care teams.

When rating your level of agreement, consider that the **final list of competency statements should capture the full range of skills and abilities that an entry-level physiotherapist would bring to a primary care practice setting**. Understanding that in clinical practice, how these competencies are enacted in a particular practice, will be dependent on the interprofessional team as a whole, and their collective skill set. For example, a physiotherapist who works in a large urban primary care setting with a nurse, dietitian and pharmacist, may work within a more focused scope of practice, as team members determine who would be best to perform a given task. In comparison, a physiotherapist who practices in a remote community, with fewer or less consistent interprofessional team members, may practice to a fuller scope, as there are less shared competencies amongst the team.

In Round 2, you will be asked to **rate your level of agreement** with each of the modified competency statements. If applicable, include comments or suggestions about how you would modify the statement to better reflect the competencies held by **all entry-level physiotherapists**.

You could either rephrase the statement or tell us what should be added, what should be removed or what should be changed. For example, in a competency statement about treatment modalities, you may agree with most of the statement, but think that not all entry-level physiotherapists would be proficient with one of the modalities mentioned. In the comments section you could write “*remove XX modality*” or you could rewrite the statement yourself with the modality removed. If you feel a competency statement should be added, please include it in your comments.

**Feedback from Round 1:**

Along with each of the following competency statements, you will find your previous rating of agreement, as well as the median (central tendency) and range (variability) of scores from the entire physiotherapist expert panel.

Each response from the Likert scale was given a value between 1-5.

Strongly agree (1), Agree (2), Neutral (3), Disagree (4), Strongly disagree (5)

**The median** of responses is the middle value when all the responses are arranged in order. For example, if 3 people voted strongly agree, 3 voted agree and 2 voted disagree, and 1 voted strongly disagree, the central tendency or median would be the middle value (**Example A**: median= 2 or Agree).

**Example A:**

1 1 1 2 2 2 3 3 4 4 5

Median= Agree

**Range,** on the other hand, shows the variability in responses for each statement. For example, if everyone strongly agreed or agreed with a competency statement, the range would be small, meaning the range of responses had low variability (**Example B**: 2-1).

**Example B:**

1 1 1 1 1 2 2 2 2 2 2

Highest value – lowest value

Range = Agree – Strongly agree

However, if most everyone strongly agreed and only one person strongly disagreed the range would be large, meaning the range of responses was highly variable (**Example C**: 5-1).

**Example C:**

1 1 1 1 1 1 1 5

Highest value – lowest value

Range = Strongly disagree – Strongly agree

_____________________________________________________________________

Domain: PHYSIOTHERAPY EXPERTISE

*Ensures physical and emotional safety of client*

Please **rate your level of agreement** with the following competency statements related to the **skills and** **abilities held by ALL licensed entry-level physiotherapists.**

If applicable, include comments or suggestions about how you would modify the statement.

**Median:** The middle value when all the responses are arranged in order

**Range:** The variability in responses for each statement

1. **Identifies client-specific precautions, contraindications and risks to physical activity participation from sensory, motor or autonomic neuropathies, retinopathy, nephropathy, active or previous history of foot ulcer, or pregnancy related complications in women with gestational diabetes**

Your previous rating:

Median: Agree (2)

Range: Disagree (4) - Strongly agree (1)

[] [] [] [] []

Strongly agree Agree Neutral Disagree Strongly disagree

Comments: [if selected strongly agree]

Please explain your selection: [if selected any other response]

1. **Identifies need for and is able to perform a foot assessment including peripheral circulation, sensory testing and skin integrity to ensure client's safety and comfort when participating in physical activity for diabetes management**

Your previous rating:

Median: Agree (2)

Range: Neutral (3) - Strongly agree (1)

[] [] [] [] []

Strongly agree Agree Neutral Disagree Strongly disagree

Comments: [if selected strongly agree]

Please explain your selection: [if selected any other response]

1. **Identifies and responds appropriately to non-glycemic related adverse responses to physical activity interventions for diabetes management**

Your previous rating:

Median: Strongly agree (1)

Range: Disagree (4) - Strongly agree (1)

[] [] [] [] []

Strongly agree Agree Neutral Disagree Strongly disagree

Comments: [if selected strongly agree]

Please explain your selection: [if selected any other response]

_____________________________________________________________________

Domain: PHYSIOTHERAPY EXPERTISE (continued)

*Conducts client assessment*

Please **rate your level of agreement** with the following competency statements related to the **skills and** **abilities held by ALL licensed entry-level physiotherapists.**

If applicable, include comments or suggestions about how you would modify the statement.

**Median:** The middle value when all the responses are arranged in order

**Range:** The variability in responses for each statement

1. **Interviews clients living with type 1, type 2 or gestational diabetes to obtain relevant information about diabetes, other health conditions, and personal and environmental factors relevant to physical activity for diabetes management**

Your previous rating:

Median: Strongly agree (1)

Range: Disagree (4) - Strongly agree (1)

[] [] [] [] []

Strongly agree Agree Neutral Disagree Strongly disagree

Comments: [if selected strongly agree]

Please explain your selection: [if selected any other response]

1. **Interviews clients to determine their personal health literacy, current self-management skills and stage of behavior change, as it relates to physical activity, and adjusts assessment, treatment plan and self-management education and support accordingly**

*Personal Health Literacy: "is the degree to which individuals have the ability to find, understand, and use information and services to inform health related decisions and actions for themselves and others" ^1^*

Your previous rating:

Median: Agree (2)

Range: Neutral (3) - Strongly agree (1)

[] [] [] [] []

Strongly agree Agree Neutral Disagree Strongly disagree

Comments: [if selected strongly agree]

Please explain your selection: [if selected any other response]

1. **Identifies risk factors such as low physical activity level, comorbidities, smoking, nutrition, and alcohol/drug use that place pre-diabetes and diabetes populations at high risk for developing diabetes or developing diabetes related complications, respectively**

Your previous rating:

Median: Agree (2)

Range: Neutral (3) - Strongly agree (1)

[] [] [] [] []

Strongly agree Agree Neutral Disagree Strongly disagree

Comments: [if selected strongly agree]

Please explain your selection: [if selected any other response]

1. **Identifies wounds or Charcot foot that may benefit from referral to other services, and advises client's accordingly**

Your previous rating:

Median: Strongly agree (1)

Range: Neutral (3) - Strongly agree (1)

[] [] [] [] []

Strongly agree Agree Neutral Disagree Strongly disagree

Comments: [if selected strongly agree]

Please explain your selection: [if selected any other response]

1. **Interprets assessment findings and develops a physiotherapy diagnosis and working prognosis in order to design appropriate physical activity intervention for diabetes management**

*(Physiotherapy diagnosis: A conclusion about physical function based on a subjective and objective assessment and analysis by a physiotherapist to investigate the cause or nature of a client’s condition or problem)^2^*

Your previous rating:

Median: Strongly agree (1)

Range: Neutral (3) - Strongly agree (1)

[] [] [] [] []

Strongly agree Agree Neutral Disagree Strongly disagree

Comments: [if selected strongly agree]

Please explain your selection: [if selected any other response]

_____________________________________________________________________

Domain: PHYSIOTHERAPY EXPERTISE (continued)

*Develops, implements, monitors and evaluates an intervention plan*

Please **rate your level of agreement** with the following competency statements related to the **skills and** **abilities held by ALL licensed entry-level physiotherapists.**

If applicable, include comments or suggestions about how you would modify the statement.

**Median:** The middle value when all the responses are arranged in order

**Range:** The variability in responses for each statement

1. **Identifies strategies to manage the hypoglycemic effect of physical activity for clients who use insulin or hyperglycemic medications with a risk of hypoglycemia including, treating acute hypoglycemia and collaboration with other primary care team members for insulin adjustments as appropriate**

Your previous rating:

Median: Agree (2)

Range: Disagree (4) - Strongly agree (1)

[] [] [] [] []

Strongly agree Agree Neutral Disagree Strongly disagree

Comments: [if selected strongly agree]

Please explain your selection: [if selected any other response]

1. **Supports clients with comorbidities to perform physical activity for diabetes management through therapeutic interventions which could include oxygen titration, breathing strategies, energy conservation, pelvic floor exercises, wound care, sensory training/retraining, electrophysical agents, spinal/peripheral joint mobilizations, neurodynamic techniques, and wheelchair skills**

Your previous rating:

Median: Agree (2)

Range: Neutral (3) - Strongly agree (1)

[] [] [] [] []

Strongly agree Agree Neutral Disagree Strongly disagree

Comments: [if selected strongly agree]

Please explain your selection: [if selected any other response]

1. **Plans, delivers and evaluates group physical activity programming for diabetes management when appropriate, given available resources**

Your previous rating:

Median: Strongly agree (1)

Range: Strongly disagree (5) - Strongly agree (1)

[] [] [] [] []

Strongly agree Agree Neutral Disagree Strongly disagree

Comments: [if selected strongly agree]

Please explain your selection: [if selected any other response]

_____________________________________________________________________

Domain: SCHOLARSHIP

Please **rate your level of agreement** with the following competency statements related to the **skills and** **abilities held by ALL licensed entry-level physiotherapists.**

If applicable, include comments or suggestions about how you would modify the statement.

**Median:** The middle value when all the responses are arranged in order

**Range:** The variability in responses for each statement

1. **Able to access and critically appraise emerging information about physical activity and diabetes and determine potential for applicability in primary care settings**

Your previous rating:

Median: Agree (2)

Range: Neutral (3) - Strongly agree (1)

[] [] [] [] []

Strongly agree Agree Neutral Disagree Strongly disagree

Comments: [if selected strongly agree]

Please explain your selection: [if selected any other response]

_____________________________________________________________________

Domain: PROFESSIONALISM

Please **rate your level of agreement** with the following competency statements related to the **skills and** **abilities held by ALL licensed entry-level physiotherapists.**

If applicable, include comments or suggestions about how you would modify the statement.

**Median:** The middle value when all the responses are arranged in order

**Range:** The variability in responses for each statement

1. **Has been granted a "physiotherapist" designation by their provincial regulatory body which, through delegated authority from the provincial government, ensures ethical, competency and professional standards of practice are maintained using mechanisms including a public registry of individual physiotherapists and a robust complaint, investigative and disciplinary program with input from members of the public**

Your previous rating:

Median: Strongly agree (1)

Range: Neutral (3) - Strongly agree (1)

** We are suggesting that the above competency statement be removed from the list of competency statements, as it is not specific to physical activity interventions for diabetes management in primary care. The information would, instead, be included as part of background information accompanying the referral pathway tool as it is a differentiating factor between physiotherapists and kinesiologists. Please rate your level of agreement with this decision below.**

[] [] [] [] []

Strongly agree Agree Neutral Disagree Strongly disagree

Comments: [if selected strongly agree]

Please explain your selection: [if selected any other response]

**14. Recognizes and addresses real, potential or perceived conflicts of interest with pharmaceutical companies and/or fitness facilities/vendors when providing physical activity interventions, education or recommendations to clients**

Your previous rating:

Median: Agree (2)

Range: Disagree (4) - Strongly agree (1)

[] [] [] [] []

Strongly agree Agree Neutral Disagree Strongly disagree

Comments: [if selected strongly agree]

Please explain your selection: [if selected any other response]

**Do you have any additional comments to share?**

___________________________________________________________________________________________________________________________________________________________

1. Centers for Disease Control and Prevention. (2022, February 2). Health Literacy. <https://www.cdc.gov/healthliteracy/learn/index.html>

2. National Physiotherapy Advisory Group. (2017). *Competency profile for physiotherapists in Canada*. <https://www.peac-aepc.ca/pdfs/Resources/Competency%20Profiles/Competency%20Profile%20for%20PTs%202017%20EN.pdf>

**This is the end of the survey.** 
 
Once you click submit, you will not be able to return to the survey to make any further change to your answers

**Thank you for taking the time to complete the survey and for your collaboration in this research project!**
