# Supplementary material for: Referral pathway and competency profiles of primary care physiotherapists and kinesiologists for physical activity interventions for diabetes: a modified Delphi study
Source: BMC Prim Care. 2024 Oct 15;25:368. doi: 10.1186/s12875-024-02611-1 (PMC11479570; doi:10.1186/s12875-024-02611-1)
Supplement: Supplementary file 4 — Additional file 4. Delphi survey. Round 2 (kinesiology). [file 12875_2024_2611_MOESM4_ESM.docx]

**Kinesiology Delphi Round Two Survey**

The following survey consists of 9 proposed competency statements for **entry-level kinesiologists who are affiliated with the Canadian Kinesiology Alliance (CKA) related to physical activity intervention for diabetes care in primary care settings.**

Following Round 1, any competency statements that all or most of the expert kinesiologist panel agreed upon have been removed from the Round 2 survey. Those that did not achieve consensus and therefore need further review have been included in this survey.

The outcome of this project will be a referral pathway tool for diabetes care by exercise specialists, for use by interprofessional primary care teams. **An expert panel of physiotherapists has completed a similar survey with their own set of competencies.**

When rating your level of agreement, consider that the final list of competency statements should **represent the skills and abilities that ALL entry-level kinesiologists (affiliated with the CKA) bring to a primary care setting without additional education/training.** For example, when rating, you should not consider the skills and abilities acquired through extra certifications such as, Canadian Society of Exercise Physiology (CSEP) Clinical Exercise Physiologist (CEP) certification.

In Round 2, you will be asked to **rate your level of agreement** with each of the modified competency statements. If applicable, include comments or suggestions about how you would modify the statement to better reflect the competencies held by **all entry-level CKA kinesiologists**.

You could either rephrase the statement or tell us what should be added, what should be removed or what should be changed. For example, in a competency statement about treatment modalities, you may agree with most of the statement, but think that not all entry-level kinesiologists would be proficient with one of the modalities mentioned. In the comments section you could write “*remove XX modality*” or you could rewrite the statement yourself with the modality removed. If you feel a competency statement should be added, please include it in your comments.

The [*Canadian Kinesiology Alliance (CKA) Competency Profile*](https://www.cka.ca/en/competency-profile-competency-required) outlines the "knowledge and skills an entry-level practitioner should possess" at the point of entry-to-practice^2^.

**Feedback from Round 1:**

Along with each of the following competency statements, you will find your previous rating of agreement, as well as the median (central tendency) and range (variability) of scores from the entire kinesiologist expert panel.

Each response from the Likert scale was given a value between 1-5.

Strongly agree (1), Agree (2), Neutral (3), Disagree (4), Strongly disagree (5)

**The median** of responses is the middle value when all the responses are arranged in order. For example, if 3 people voted strongly agree, 3 voted agree and 2 voted disagree, and 1 voted strongly disagree, the central tendency or median would be the middle value (**Example A**: median= 2 or Agree).

**Example A:**

1 1 1 2 2 2 3 3 4 4 5

Median= Agree

**Range,** on the other hand, shows the variability in responses for each statement. For example, if everyone strongly agreed or agreed with a competency statement, the range would be small, meaning the range of responses had low variability (**Example B**: 2-1).

**Example B:**

1 1 1 1 1 2 2 2 2 2 2

Highest value – lowest value

Range = Agree – Strongly agree

However, if most everyone strongly agreed and only one person strongly disagreed the range would be large, meaning the range of responses was highly variable (**Example C**: 5-1).

**Example C:**

1 1 1 1 1 1 1 5

Highest value – lowest value

Range = Strongly disagree – Strongly agree

_____________________________________________________________________

Domain: KINESIOLOGY EXPERTISE

*Ensures physical and emotional safety of client*

Please **rate your level of agreement** with the following competency statements related to the **skills and** **abilities held by ALL CKA affiliated entry-level kinesiologists.**

If applicable, include comments or suggestions about how you would modify the statement.

**Median:** The middle value when all the responses are arranged in order

**Range:** The variability in responses for each statement

1. **Identifies client-specific precautions, contraindications and risks to physical activity participation from preproliferative, proliferative retinopathy, autonomic neurological dysfunction, foot ulcer or pregnancy related complications in women with gestational diabetes**

Your previous rating:

Median: Agree (2)

Range: Neutral (3) - Strongly agree (1)

[] [] [] [] []

Strongly agree Agree Neutral Disagree Strongly disagree

Comments: [if selected strongly agree]

Please explain your selection: [if selected any other response]

1. **Identifies signs and symptoms of hypoglycemic and hyperglycemic emergencies in response to physical activity and takes appropriate action**

Your previous rating:

Median: Agree (2)

Range: Neutral (3) - Strongly agree (1)

[] [] [] [] []

Strongly agree Agree Neutral Disagree Strongly disagree

Comments: [if selected strongly agree]

Please explain your selection: [if selected any other response]

_____________________________________________________________________

Domain: KINESIOLOGY EXPERTISE (continued)

*Conducts client assessment*

Please **rate your level of agreement** with the following competency statements related to the **skills and** **abilities held by ALL CKA affiliated entry-level kinesiologists.**

If applicable, include comments or suggestions about how you would modify the statement.

**Median:** The middle value when all the responses are arranged in order

**Range:** The variability in responses for each statement

1. **Selects and performs appropriate tests and measures to identify current fitness level and potential barriers to physical activity for diabetes management including pelvic ligament laxity, diastasis recti, impaired skin integrity, respiratory, vascular or neurological impairments, cognitive or mental health disorders, musculoskeletal injury or chronic pain**

Your previous rating:

Median: Agree (2)

Range: Strongly disagree (5) - Strongly agree (1)

[] [] [] [] []

Strongly agree Agree Neutral Disagree Strongly disagree

Comments: [if selected strongly agree]

Please explain your selection: [if selected any other response]

_____________________________________________________________________

Domain: KINESIOLOGY EXPERTISE (continued)

*Develops, implements, monitors and evaluates an intervention plan*

Please **rate your level of agreement** with the following competency statements related to the **skills and** **abilities held by ALL CKA affiliated entry-level kinesiologists.**

If applicable, include comments or suggestions about how you would modify the statement.

**Median:** The middle value when all the responses are arranged in order

**Range:** The variability in responses for each statement

1. **Identifies strategies to manage the hypoglycemic effect of physical activity for clients who use insulin or anti-hyperglycemic medications with a risk of hypoglycemia**

Your previous rating:

Median: Agree (2)

Range: Neutral (3) - Strongly agree (1)

[] [] [] [] []

Strongly agree Agree Neutral Disagree Strongly disagree

Comments: [if selected strongly agree]

Please explain your selection: [if selected any other response]

1. **Supports clients with comorbidities to perform physical activity for diabetes management through therapeutic interventions including ice, heat, exercise, and taping.**

Your previous rating:

Median: Neutral (3)

Range: Strongly disagree (5) - Agree (2)

[] [] [] [] []

Strongly agree Agree Neutral Disagree Strongly disagree

Comments: [if selected strongly agree]

Please explain your selection: [if selected any other response]

_____________________________________________________________________

Domain: COLLABORATION

Please **rate your level of agreement** with the following competency statements related to the **skills and** **abilities held by ALL CKA affiliated entry-level kinesiologists.**

If applicable, include comments or suggestions about how you would modify the statement.

**Median:** The middle value when all the responses are arranged in order

**Range:** The variability in responses for each statement

1. **Fosters collaborative relationships with interprofessional diabetes care team**

Your previous rating:

Median: Strongly agree (1)

Range: Neutral (3) - Strongly agree (1)

[] [] [] [] []

Strongly agree Agree Neutral Disagree Strongly disagree

Comments: [if selected strongly agree]

Please explain your selection: [if selected any other response]

_____________________________________________________________________

Domain: PROFESSIONALISM

Please **rate your level of agreement** with the following competency statements related to the **skills and** **abilities held by ALL CKA affiliated entry-level kinesiologists.**

If applicable, include comments or suggestions about how you would modify the statement.

**Median:** The middle value when all the responses are arranged in order

**Range:** The variability in responses for each statement

1. **Complies with the code of ethics established by their professional body and is committed to continuing competency**

Your previous rating:

Median: Strongly agree (1)

Range: Neutral (3) - Strongly agree (1)

[] [] [] [] []

Strongly agree Agree Neutral Disagree Strongly disagree

Comments: [if selected strongly agree]

Please explain your selection: [if selected any other response]

1. **Recognizes and addresses conflicts of interest with pharmaceutical companies and fitness facilities/vendors**

Your previous rating:

Median: Strongly agree (1)

Range: Neutral (3) - Strongly agree (1)

[] [] [] [] []

Strongly agree Agree Neutral Disagree Strongly disagree

Comments: [if selected strongly agree]

Please explain your selection: [if selected any other response]

1. **Recognizes, explores and acknowledges the relationship between the legacy of colonization and current high rates of diabetes amongst Indigenous peoples**

Your previous rating:

Median: Neutral (3)

Range: Neutral (3) - Strongly agree (1)

[] [] [] [] []

Strongly agree Agree Neutral Disagree Strongly disagree

Comments: [if selected strongly agree]

Please explain your selection: [if selected any other response]

**Do you have any additional comments to share?**

___________________________________________________________________________________________________________________________________________________________

**This is the end of the survey.** 
 
**Once you click submit, you will not be able to return to the survey to make any further change to your answers.**

**Thank you for taking the time to complete the survey and for your collaboration in this research project!**
