# Supplementary material for: Referral pathway and competency profiles of primary care physiotherapists and kinesiologists for physical activity interventions for diabetes: a modified Delphi study
Source: BMC Prim Care. 2024 Oct 15;25:368. doi: 10.1186/s12875-024-02611-1 (PMC11479570; doi:10.1186/s12875-024-02611-1)
Supplement: Supplementary file 5 — Additional file 5. Focus group interview guide. Semi-structure interview discussion guide (physiotherapy and kinesiology). [file 12875_2024_2611_MOESM5_ESM.docx]

**Physiotherapy Focus Group Discussion Guide**

**Welcome**

**Introduction of facilitator and notetaker**

Thank you for your participation in this study thus far and for agreeing to participate in the focus group today. To review, the purpose of this study is to establish the common and distinct competencies of entry-level physiotherapists and kinesiologists in physical activity for diabetes in primary care settings and develop a referral pathway tool for diabetes care by exercise specialists for use by interprofessional primary care teams.

The purpose of the focus group today is to determine how well the final competency statements represent entry-level physiotherapist competencies in physical activity for diabetes in primary care and to determine the validity of the referral pathway tool and its usefulness in practice.

This focus group will last for 90 minutes and both audio and video will be recorded as a record of today’s discussion.

**Ground rules and note about confidentiality.**

**QUESTIONS**

**Introductions**

1. We will just start off with some introductions. We will go around the screen and ask everyone to give your first name and your current role.

**Competency Statements**

We are going to talk about the competency statements that were sent out on [insert date here]. We will talk about them generally to begin with and then go through them one by one to get feedback.

1. What was your overall impression of the competency statements?

Let’s look at each of these statements one at a time.

[Start screen sharing first competency statement]

1. Thinking about entry-level physiotherapists in primary care settings, how well does this competency statement reflect their knowledge and skills related to physical activity in diabetes care?

[Next competency statement]

1. How well does this statement reflect their knowledge and skills?
2. Repeat for remainder of competency statements.
3. Is there anything else you would add?
4. Is there anything else you would change?

**Referral Pathway Tool**

Moving on to the referral pathway tool

[screen share referral pathway tool]

1. What was your overall impression of the referral pathway tool?
2. What do you like about the referral pathway tool?
3. Does it differentiate between physiotherapists and kinesiologists?
4. Is it easy to follow?
5. If you were making this tool, what would you do differently?
6. How do you think this tool would be received in primary care practices?
7. How do you think it will be used?
8. Which team members do you think will use it?
9. What do you think might be some challenges to using this tool in primary care?

**Closing**

Thanks for the great discussion. As we are coming to the end of our time together, I want to bring us back to the purpose of this project.

The objectives of this study were to define the competencies of entry level physiotherapists and kinesiologists in physical activity for diabetes management, and then develop a practical referral pathway tool that could be used by primary care teams with the purpose of promoting effective interprofessional collaboration for diabetes care.

The goal of this focus group was to find out how well the final competency statements reflect your expert opinion about the competencies of entry-level physiotherapists related to physical activity in diabetes care and the accuracy and usefulness of the referral pathway tool.

1. With that in mind, is there anything else we did not cover that we should have talked about today?

**Kinesiology Focus Group Discussion Guide**

**Welcome**

**Introduction of facilitator and notetaker**

Thank you for your participation in this study thus far and for agreeing to participate in the focus group today. To review, the purpose of this study is to establish the common and distinct competencies of entry-level physiotherapists and kinesiologists in physical activity for diabetes in primary care settings and develop a referral pathway tool for diabetes care by exercise specialists for use by interprofessional primary care teams.

The purpose of the focus group today is to determine how well the final competency statements represent entry-level kinesiologist competencies in physical activity for diabetes in primary care and to determine the validity of the referral pathway tool and its usefulness in practice.

This focus group will last for 90 minutes and both audio and video will be recorded as a record of today’s discussion.

**Ground rules and note about confidentiality**

**QUESTIONS**

**Introductions**

1. We will just start off with some introductions. We will go around the screen and ask everyone to give your first name and your current role.

**Competency Statements**

We are going to talk about the competency statements that were sent out on [insert date here]. We will talk about them generally to begin with and then go through them one by one to get feedback.

1. What was your overall impression of the competency statements?

Let’s look at each of these statements one at a time.

[Start screen sharing first competency statement]

1. Thinking about entry-level kinesiologists in primary care settings, how well does this competency statement reflect their knowledge and skills related to physical activity in diabetes care?

[Next competency statement]

1. How well does this statement reflect their knowledge and skills?
2. Repeat for remainder of competency statements
3. Is there anything else you would add?
4. Is there anything else you would change?

**Referral Pathway Tool**

Moving on to the referral pathway tool

[screen share referral pathway tool]

1. What was your overall impression of the referral pathway tool?
2. What do you like about the referral pathway tool?
3. Does it differentiate between physiotherapists and kinesiologists?
4. Is it easy to follow?
5. If you were making this tool, what would you do differently?
6. How do you think this tool would be received in primary care practices?
7. How do you think it will be used?
8. Which team members do you think will use it?
9. What do you think might be some challenges to using this tool in primary care?

**Closing**

Thanks for the great discussion. As we are coming to the end of our time together, I want to bring us back to the purpose of this project.

The objectives of this study were to define the competencies of entry level physiotherapists and kinesiologists in physical activity for diabetes management, and then develop a practical referral pathway tool that could be used by primary care teams with the purpose of promoting effective interprofessional collaboration for diabetes care.

The goal of this focus group was to find out how well the final competency statements reflect your expert opinion about the competencies of entry-level kinesiologist related to physical activity in diabetes care and the accuracy and usefulness of the referral pathway tool.

1. With that in mind, is there anything else we did not cover that we should have talked about today?
