# Supplementary material for: Referral pathway and competency profiles of primary care physiotherapists and kinesiologists for physical activity interventions for diabetes: a modified Delphi study
Source: BMC Prim Care. 2024 Oct 15;25:368. doi: 10.1186/s12875-024-02611-1 (PMC11479570; doi:10.1186/s12875-024-02611-1)
Supplement: Supplementary file 6 — Additional file 6. Fig. 1: Physiotherapy competency profile. [file 12875_2024_2611_MOESM6_ESM.pdf]

Physical Activity for Diabetes Management in Primary Care
Competency Profile for Entry-Level Physiotherapists

The following competency profile describes the abilities of entry-level physiotherapists related to the specific clinical context of physical activity interventions for diabetes management in primary care settings.

Physiotherapists are regulated by licensing bodies who ensure ethical, competency and professional standards of practice are maintained using mechanisms including:

- public registry of individual physiotherapists
- a robust complaint, investigative and disciplinary program with input from members of the public

The entry-level physiotherapist has completed their education, is licensed by a provincial regulatory body to practice in Canada as a clinician and is at the starting point of continuing professional development.

The competency statements in this document are intended to provide guidance in several ways to all members of interprofessional primary care teams, including:

- informing decision making related to the inclusion of physiotherapists on primary care teams for diabetes management,
- enhancing interprofessional collaboration by improving role clarity of physiotherapists, and
- encouraging referrals to physiotherapists for physical activity intervention for diabetes prevention and management.

Competency Profile

This competency profile relates to physical activity interventions for individuals living with pre-diabetes, type 1 diabetes, type 2 diabetes and gestational diabetes.

Domain 1: PHYSIOTHERAPY EXPERTISE

Ensures physical and emotional safety of client

As experts in exercise, mobility and function, physiotherapists ensure the physical and emotional safety of clients while participating in physical activity by using clinical reasoning that integrates unique knowledge and skills to enhance the health and wellbeing of their clients.

1. Identifies client-specific precautions, contraindications and risks to physical activity participation from acute hyperglycemia, hypoglycemia or pseudo-hypoglycemia
2. Identifies client-specific precautions, contraindications and risks to physical activity participation from cardiovascular complications, sensory, motor or autonomic neuropathies, falls risk, retinopathy, nephropathy, active or previous history of foot ulcer, or pregnancy related complications in women with gestational diabetes
3. Identifies client-specific precautions, contraindications and risks to physical activity participation from non-diabetes related comorbidities in people living with diabetes
4. Monitors relevant parameters including blood pressure, oximetry, heart rate, and respiratory rate during assessment and physical activity intervention that enhances the client's safety and comfort
5. Identifies need for and makes recommendations for ambulatory, assistive, adaptive, and protective devices (including footwear) to support client's safety and comfort when participating in physical activity
6. Identifies need for and is able to perform a foot assessment including peripheral circulation, sensory testing, skin integrity and biomechanics of the foot and lower extremity to ensure client's safety and comfort when participating in physical activity for diabetes management
7. Identifies signs and symptoms of hypoglycemic and hyperglycemic emergencies in response to physical activity and takes appropriate action
8. Identifies and responds appropriately to non-glycemic related adverse responses to physical activity interventions for diabetes management

Conducts client assessment

As experts in exercise, mobility and function, physiotherapists conduct comprehensive subjective and objective assessments that identify barriers and facilitators to physical activity interventions for diabetes management

9. Interviews clients to obtain relevant information about diabetes, other health conditions, and personal and environmental factors relevant to physical activity for diabetes management
10. Interviews clients to determine their personal health literacy, current self-management skills and current state of readiness, as it relates to physical activity, and adjusts assessment, treatment plan and self-management education and support accordingly
11. Obtains information about client's status from lab work, diagnostic imaging, electrocardiogram or pulmonary function test results relevant to physical activity for diabetes management
12. Identifies risk factors such as sedentary behavior, low physical activity levels, comorbidities, smoking, nutrition, and alcohol/drug use that place pre-diabetes and diabetes populations at high risk for developing diabetes or developing diabetes related complications, respectively
13. As a primary care practitioner, identifies yellow, orange or red flags and makes appropriate referrals to other team members and/or makes appropriate changes to assessment and treatment plans for physical activity interventions for diabetes care
14. Identifies acute and chronic changes such as inflammation, wounds or deformities that may indicate inappropriate fitting footwear or assistive, adaptive and protective devices and may benefit from referral to other services, and advises clients accordingly
15. Selects and performs appropriate tests and measures to identify current fitness level and potential barriers to physical activity for diabetes management related to impaired skin integrity, chronic pain, vascular, respiratory, neurological, or musculoskeletal impairments
16. Identifies cognitive, mental health, social or financial barriers to physical activity for diabetes management that may benefit from referral to other services
17. Interprets assessment findings and develops a physiotherapy diagnosis and working prognosis in order to design appropriate physical activity intervention for diabetes management

Develops, implements, monitors and evaluates an intervention plan

As experts in exercise, mobility and function, physiotherapists integrate unique knowledge, skills and attitudes to provide client centered physical activity interventions

18. Establishes a physical activity goal for diabetes management in collaboration with client, and develops an intervention plan appropriate to the client's goals, current state of readiness, current health status and that is in alignment with the client's resources and abilities
19. Educates clients about the benefits and risks of various physical activities with consideration for individual's existing comorbidities
20. Implements and monitors customized physical activity intervention including aerobic, strength, flexibility and/or balance exercises designed to optimize glycemic control, cardiorespiratory fitness, diabetes complication risk and/or quality of life
21. Provides self-management education and support to clients, families and communities to support physical activity for diabetes management including footwear and nutrition.
22. Identifies strategies to manage the hypoglycemic effect of physical activity for clients who use insulin or anti-hyperglycemic medications with a risk of hypoglycemia including, treating acute hypoglycemia and collaboration with other primary care team members for insulin adjustments as appropriate
23. Supports clients with comorbidities to perform physical activity for diabetes management through therapeutic interventions which could include oxygen titration, breathing strategies, energy conservation, pelvic floor exercises, sensory training/retraining, falls prevention, electrophysical agents, spinal/peripheral joint mobilizations, neurodynamic techniques, and wheelchair skills
24. Monitors skin, wounds, blisters and scars during physical activity for diabetes management and responds appropriately with basic wound hygiene, self-management education, and if needed, referral to other services
25. Monitors client's physical, cardiovascular, respiratory, neurological and metabolic response to physical activity intervention for diabetes management, reassesses client's needs and modifies physical activity intervention plan as indicated
26. Identifies opportunities for group physical activity programming for diabetes management and plans, delivers and evaluates physical activity programs when appropriate, given available resources

Domain 2: COLLABORATION

As collaborators, physiotherapists use effective communication strategies to exchange information, enhance therapeutic and professional relationships, and work effectively with others to provide inter- and intraprofessional care that is effective and sustainable.

27. Employs a client-centered approach by acting in a manner that respects client uniqueness, diversity and autonomy, with the client, their family and/or community as key members of the diabetes management care team
28. Facilitates collaborative relationships with interprofessional diabetes care team
29. Provides services that balance waitlists, client needs and available resources including delegating care to and supervising personnel involved in physiotherapy service delivery for diabetes care
30. Identifies the learning needs related to physical activity and diabetes management of other healthcare providers and contributes to and assesses the effectiveness of learning activities

Domain 3: SCHOLARSHIP

As scholars, physiotherapists demonstrate a commitment to excellence in practice through continuous learning, the education of others, and the evaluation of evidence.

31. Able to access and critically appraise emerging information about physical activity and diabetes and determine potential for applicability in primary care settings
32. Uses a structured evidence-informed approach incorporating best available evidence, client context and personal knowledge and experience into clinical decision making for diabetes care

Domain 4: PROFESSIONALISM

As autonomous, self-regulated professionals, physiotherapists are committed to maintaining high standards of behavior and working in the best interest of clients and society including, envisioning and advocating for a health system that enhances the wellbeing of society

33. Advocates for the value of physiotherapy services to overcome barriers and facilitate improved physical activity interventions in diabetes care in primary care settings
34. Recognizes and addresses real, potential or perceived conflicts of interest with pharmaceutical companies and/or fitness facilities/vendors when providing physical activity interventions, education or recommendations to clients
35. Demonstrates awareness of the social determinants of health in diabetes management and advocates for physical activity opportunities and support that are sustainable, socially, and culturally appropriate and geographically accessible to clients year round

36. Recognizes that the cause of current high rates of diabetes amongst Indigenous peoples is complex and is strongly associated with the legacy of colonization and provides care that is congruent with Indigenous social and cultural contexts.
37. Incorporates a purposeful process of self-reflection and learning into one's clinical practice to promote cultural safety by, recognizing one's own assumptions and respecting an Indigenous person's concept of health and their preferences or barriers to engaging in clinical interactions or traditional healing resources
38. Understands and works within physiotherapy scope of practice and personal level of competence in diabetes management as required by provincial regulatory licensing body

Definitions

Ambulatory devices: *canes, crutches, walkers, walking poles, wheelchairs*[1]
Assistive, adaptive, protective devices: *splints, taping/wrapping, bandaging, braces, orthotics, garments, collars*[1]
Diversity: *refers to variation among people including, but not limited to, variation based upon factors such as race, ethnicity, colour, religion, age, sex, sexual orientation, marital status, family status, and disability*[1]
Personal health literacy: *"is the degree to which individuals have the ability to find, understand, and use information and services to inform health related decisions and actions for themselves and others"* [2]
Personnel involved in physiotherapy service delivery: *includes support personnel, assistants, volunteers, and other healthcare providers, who may provide physiotherapy services under the direction and supervision of a physiotherapist*[1]
Physiotherapy diagnosis: *a conclusion about physical function based on a subjective and objective assessment and analysis by a physiotherapist to investigate the cause or nature of a client's condition or problem*[1]
Physiotherapy services: *services provided by or under the direction of a physiotherapist. This includes client assessment and intervention, and related communication with and reporting to various parties for the purposes of delivering client care*[1]
Yellow flags: *maladaptive pain coping strategies*
Orange flags: *psychiatric symptoms*
Red flags: *signs of serious pathology*[3]

Acknowledgements

A diverse group of subject matter experts from Alberta, Manitoba and Nova Scotia contributed content expertise in the development of this competency profile for physiotherapists delivering physical activity interventions for diabetes management in primary care settings. Their thoughtful contributions over a 5-month period have enhanced the confidence that this competency profile accurately reflects the competencies of physiotherapists in primary care settings.

Competency Profile Development

Following best practices for gathering expert knowledge, the Delphi method was used to establish this competency profile, followed by a focus group to validate the document.

The National Physiotherapy Advisory Group's (NPAG) Competency Profile for Physiotherapists in Canada[1] was used as the basic framework for the initial competency statements. Informed by the literature on best practices in diabetes care, including the Diabetes Canada Clinical Practice Guidelines[4], NPAG competency statements that were relevant to physical activity interventions for diabetes care in primary care settings were modified to reflect the intervention, patient population and clinical context. The modified competencies primarily fell into 4 of the 7 domains: physiotherapy expertise, collaboration, scholarship, and professionalism.

Eleven subject matter experts (clinicians, researchers and educators), recruited from across Canada, revised the competency statements through two iterative Delphi survey rounds. The draft of competencies was validated through focus groups consisting of a smaller group of the same subject matter experts.
To see the full competency profile for physiotherapists across all practice settings and interventions see NPAG Competency Profile for Physiotherapists in Canada (2017).

References

1. National Physiotherapy Advisory Group. *Competency Profile for Physiotherapist in Canada*.; 2017. Accessed September 8, 2021. https://www.peac-aepc.ca/pdfs/Resources/Competency%20Profiles/Competency%20Profile%20for%20PTs%202017%20EN.pdf
2. Centers for Disease Control and Prevention. Health Literacy. Published February 2, 2022. Accessed June 6, 2022. https://www.cdc.gov/healthliteracy/learn/index.html
3. Nicholas MK, Linton SJ, Watson PJ, Main CJ. Early identification and management of psychological risk factors ("yellow flags") in patients with low back pain: A reappraisal. *Phys Ther*. 2011;91(5):737-753. doi:10.2522/PTJ.20100224
4. Diabetes Canada Clinical Practice Guidelines Expert Committee. Diabetes Canada 2018 Clinical Practice Guidelines for the Prevention and Management of Diabetes in Canada. *Can J Diabetes*. 2018;42(Suppl 1):S1-S325.
