# Supplementary material for: Referral pathway and competency profiles of primary care physiotherapists and kinesiologists for physical activity interventions for diabetes: a modified Delphi study
Source: BMC Prim Care. 2024 Oct 15;25:368. doi: 10.1186/s12875-024-02611-1 (PMC11479570; doi:10.1186/s12875-024-02611-1)
Supplement: Supplementary file 7 — Additional file 7. Fig. 2: Kinesiology competency profile. [file 12875_2024_2611_MOESM7_ESM.pdf]

Physical Activity for Diabetes Management in Primary Care
Competency Profile for Entry-Level Kinesiologists

The following competency profile describes the abilities of entry-level kinesiologists related to the specific clinical context of physical activity interventions for diabetes management in primary care settings.

Unlike most other health professions, kinesiologists are not a self-regulated profession across Canada* and therefore, the skills and abilities of individual kinesiologists may vary significantly. In the development of these competencies, we focused on kinesiologists who are affiliated with the Canadian Kinesiology Alliance (CKA). The CKA is the largest professional organization for kinesiologists in Canada and, in lieu of a regulatory body, it strives to support some of those functions for kinesiologists.

The entry-level CKA affiliated kinesiologist has completed a degree in an approved kinesiology program at a Canadian university (or equivalent) and is at the starting point of continuing professional development.

The competency statements in this document are intended to provide guidance in several ways to all members of interprofessional primary care teams, including:

- informing decision making related to the inclusion of kinesiologists on primary care teams for diabetes management,
- enhancing interprofessional collaboration by improving role clarity of kinesiologists, and
- encouraging referrals to kinesiologists for physical activity intervention for diabetes prevention and management.

NOTE: Some kinesiologists may choose to pursue additional education/training that may expand their competencies and scope of practice related to physical activity interventions for diabetes. Most notable would be the Canadian Society of Exercise Physiology (CSEP) Clinical Exercise Physiologist® certification, whose scope of practice can be found here [4]

* Ontario is the only province where Kinesiology is a regulated health profession and is regulated by the College of Kinesiologists of Ontario (COKO).

Competency Profile

This competency profile relates to physical activity management for individuals living with pre-diabetes, type 1 diabetes, and type 2 diabetes. Kinesiologists would require additional training to work with people who are pregnant.

Some competencies would require that the kinesiologist have additional training and mentorship with the primary care diabetes team to become competent.

† Additional training required for all clients living with diabetes
‡ Additional training required for clients living with type 1 diabetes only

Domain 1: KINESIOLOGY EXPERTISE

Ensures physical and emotional safety of client

Kinesiologists integrate knowledge, skills and judgment to ensure the physical and emotional safety of clients while participating in physical activity for diabetes management

1. Supports clients to participate in physical activity considering client-specific precautions, contraindications or risks related to acute hyperglycemia, hypoglycemia or pseudo-hypoglycemia†
2. Supports clients to participate in physical activity considering client-specific precautions, contraindications or risks related to comorbidities
3. Monitors relevant parameters including blood pressure, oximetry, heart rate, respiratory rate during assessment and physical activity interventions that enhances the client's safety and comfort
4. Identifies signs and symptoms of hypoglycemic and hyperglycemic emergencies in response to physical activity and takes appropriate action†
5. Identifies and responds to non-glycemic adverse responses to physical activity interventions for diabetes management with exercise modifications, education and/or consultation with appropriate health care provider

Conducts client assessment

Kinesiologists integrate knowledge, skills and judgment to conduct assessments that identify barriers and facilitators to physical activity interventions for diabetes management

6. Interviews clients living with diabetes to obtain relevant information about diabetes, other health conditions, and personal and environmental factors relevant to physical activity for diabetes management

7. Interviews clients to determine their knowledge of diabetes, current self-management skills, and current state of readiness and adjusts assessment and treatment plan accordingly
8. Identifies risk factors such as comorbidities, smoking, nutritional, alcohol/drug use, and activity level that place healthy or pre-diabetes populations at high risk for developing diabetes and those already living with diabetes, at high risk of developing diabetes related complications
9. Selects and performs appropriate tests and measures to identify current fitness levels and potential barriers to physical activity for diabetes management and makes appropriate referrals when clinically indicated
10. Able to understand, evaluate, and interpret assessment findings and referral documentation to form a clinical impression

Develops, implements, monitors and evaluates an intervention plan

Kinesiologists apply and integrate knowledge, skills and judgment to provide competent physical activity interventions for diabetes management through the continuum of care

11. Develops an intervention plan appropriate to the client's goals, current state of readiness, current health status and personal and environmental factors
12. Educates clients living with pre-diabetes and diabetes about the benefits of various physical activities
13. Implements and monitors customized physical activity intervention including aerobic, strength, flexibility and/or balance exercises designed to optimize glycemic control, cardiorespiratory fitness, diabetes complication risk and/or quality of life[‡]
14. Assists clients to develop self-management skills in physical activity and nutrition counselling to support physical activity for diabetes management
15. Identifies strategies to manage the hypoglycemic effect of physical activity for clients who use insulin or anti-hyperglycemic medications with a risk of hypoglycemia[†]
16. Supports clients with comorbidities to perform physical activity for diabetes management through appropriate modification of a therapeutic exercise program
17. Monitors client's response to physical activity intervention for diabetes management, reassesses client's needs and modifies physical activity intervention plan as indicated[‡]
18. Identifies opportunities for group physical activity programming for diabetes management and plans, delivers and evaluates programs

Domain 2: COLLABORATION

Kinesiologists use effective communication and collaboration with others to achieve common goals and enhance relationships in the provision of kinesiology services

19. Acts in a manner that respects client's diversity, autonomy and is in the best interest of the client when delivering physical activity interventions for diabetes management
20. Fosters collaborative relationships with interprofessional diabetes care team

Domain 3: SCHOLARSHIP

Kinesiologists engage in self-reflection, continuing education and development for ongoing competence

21. Able to use best practice guidelines including the interpretation and application of current evidence-based knowledge into clinical decision making for physical activity intervention in diabetes care

Domain 4: PROFESSIONALISM

Kinesiologists assume professional responsibility to provide safe, ethical and effective kinesiology services

22. Able to facilitate client access to kinesiology services and resources to physical activity interventions in diabetes care in primary care
23. Complies with the code of ethics established by their professional body and is committed to continuing competency
24. Recognizes and addresses conflicts of interest with pharmaceutical companies and fitness facilities/vendors
25. Demonstrates awareness of the social determinants of health and understands its influence on diabetes management
26. Recognizes that the cause of current high rates of diabetes amongst Indigenous peoples is complex and is strongly associated with the legacy of colonization and provides care that is congruent with Indigenous social and cultural contexts
27. Understands and works within own professional knowledge, competence, and skill set in diabetes management

Definitions

Diversity: refers to variation among people including, but not limited to, variation based upon factors such as race, ethnicity, colour, religion, age, sex, sexual orientation, marital status, family status, and disability [1]

Acknowledgements

A diverse group of subject matter experts from Alberta, Ontario and Nova Scotia contributed content expertise in the development of this competency profile for kinesiologists delivering physical activity interventions for diabetes management in primary care settings. Their thoughtful contributions over a 5-month period have enhanced the confidence that this competency profile accurately reflects the competencies of CKA affiliated kinesiologists in primary care settings.

Competency Profile Development

Following best practices for gathering expert knowledge, the Delphi method was used to establish this competency profile, followed by a focus group to validate the document.

The National Physiotherapy Advisory Group (NPAG) Competency Profile for Physiotherapists in Canada[1] was used as the basic framework for the initial competency statements. The Canadian Kinesiology Alliance Competency Profile[2], the Essential Competencies of Practice for Kinesiologists in Ontario[3] and, the literature on best practices in diabetes care, including the Diabetes Canada Clinical Practice Guidelines[5] informed modifications to the competency statements. NPAG competency statements that were relevant to physical activity interventions for diabetes care in primary care settings were modified to reflect kinesiologists and the intervention, patient population and clinical context. The modified competencies primarily fell into 4 of the 7 domains: kinesiology expertise, collaboration, scholarship, and professionalism.

Three subject matter experts (clinicians, researchers and educators), recruited from across Canada, revised the competency statements through two iterative Delphi survey rounds. The draft of competencies was validated through an interview and a focus group of the same subject matter experts.

To see the full competency profile for CKA affiliated kinesiologists across all practice settings and interventions see CKA Competency Profile (2021).
More information about the practice of kinesiology in Ontario can be found on the COKO website including the latest full competency profile at www.coko.ca.

References

1. National Physiotherapy Advisory Group. *Competency Profile for Physiotherapist in Canada*.; 2017. Accessed September 8, 2021. https://www.peac-aepc.ca/pdfs/Resources/Competency%20Profiles/Competency%20Profile%20for%20PTs%202017%20EN.pdf
2. Canadian Kinesiology Alliance. What are the requirements for affiliation? Accessed September 8, 2021. https://www.cka.ca/en/requirements-affiliation
3. College of Kinesiologists of Ontario. Essential Competencies of Practice for Kinesiologists in Ontario . Published October 2014. Accessed August 11, 2022. https://www.coko.ca/wp-content/uploads/2020/06/Essential-Competencies-of-Practice-for-Kinesiologists-in-Ontario-March-2018.pdf
4. Canadian Society for Exercise Physiology. CSEP Clinical Exercise Physiologist® Scope of Practice . Published November 2019. Accessed August 11, 2022. https://csep.ca/wp-content/uploads/2021/05/CSEP-CEP_Scope_of_Practice.pdf
5. Diabetes Canada Clinical Practice Guidelines Expert Committee. Diabetes Canada 2018 Clinical Practice Guidelines for the Prevention and Management of Diabetes in Canada. *Canadian Journal of Diabetes*. 2018;42(Suppl 1):S1-S325.
